# Structural and mechanistic insights into protective non-neutralizing antibodies targeting Crimean-Congo hemorrhagic fever virus nucleocapsid protein

**DOI:** 10.1038/s41467-026-76702-1

**Published:** 2026-08-21

**Authors:** Vanessa Moresco, Aura R. Garrison, Clarissa A. Edmundo, Collin J. Fitzpatrick, Elif Karaaslan, Scott P. Olschner, Keersten M. Ricks, Oluwadara T. Ogundare, Laliv Tadri, Brian D. Carey, Mohammad M. Sajadi, Éric Bergeron, Joseph W. Golden, Scott D. Pegan

**Affiliations:** 1 Division of Biomedical Sciences, University of California Riverside, Riverside, CA USA; 2 Virology Division, United States Army Medical Research Institute of Infectious Diseases, Fort Detrick, MD USA; 3 Chenega Professional and Technical Service, Chesapeake, VA USA; 4 Viral Special Pathogens Branch, Division of High-Consequence Pathogens and Pathology, Centers for Disease Control and Prevention, Atlanta, GA USA; 5 Diagnostic Systems Division, United States Army Medical Research Institute of Infectious Diseases, Fort Detrick, MD USA; 6 Institute of Human Virology, University of Maryland School of Medicine, Baltimore, MD USA; 7 Department of Chemistry and Biological Science, and Engineering, United States Military Academy, West Point, NY USA

**Keywords:** Antibody therapy, X-ray crystallography, Virus-host interactions

## Abstract

Crimean-Congo Hemorrhagic Fever Virus (CCHFV) is a tick-borne virus endemic to Africa, Asia, and expanding regions within Europe. With mortality rates approaching 40%, rising incidence, and no currently approved countermeasures, CCHFV is recognized as a priority public health threat. CCHFV nucleocapsid protein (NP) has long been a key target for diagnostics. Recently, NP-specific humoral responses have also been correlated with protection conferred by protective vaccines candidates. Additionally, the first non-neutralizing monoclonal antibody (mAb) 9D5 demonstrated protective efficacy against CCHFV challenge, underscoring NP as a viable antiviral target. Here, nine anti-NP mAb were utilized to identify four antigenic sites on NP as well as localize these sites to the head or stalk domains. These mAb also revealed variable levels of in vivo protection, independent from whether the epitope site is located in the head or stalk regions. Additionally, three X-ray crystallography structures were obtained that included CCHFV NP from strain Afg09-2990 in complex with the most potent mAb (9D5). This, along with additional structures of two unbound NPs, revealed structural elements critical for mAb-9D5 broad-spectrum protective characteristics. These findings provide a path towards the rapid identification of broadly protective anti-NP mAb countermeasures.

## Abstract (web summary)

This study identifies four antigenic sites on CCHFV NP, showing that protection is independent of head or stalk location. It maps the highly conserved binding epitope of mAb-9D5 in the head domain, enabling broad-spectrum efficacy and opening the door for effective combination therapies.

## Introduction

Crimean-Congo hemorrhagic fever virus (CCHFV) is an often-fatal tick-borne virus, one of over 50 members of the *Nairoviridae* family, within the genus *Orthonairovirus*^1,2^. Among all known tick-borne viruses, CCHFV has the widest geographic distribution area^2,3^. Following the range of its principal host, ticks of the genus *Hyalomma*, CCHFV occurrence stretches from western China and southern Asia to the Middle East, Eastern and Southern Europe, and Africa^3,4^. In humans, CCHFV can cause severe hemorrhagic disease with mortality rates reaching up to 40%^5,6^. CCHFV serves as the prototype member for the family *Nairoviridae*, which includes several other species capable of causing disease in humans. These comprise Dugbe virus, Issyk-kul virus, Kasokero virus, Erve virus, and the recently identified pathogenic members of the *Norwavirus* genus (Benji)^7^, and of the Tamdy (Songling and Wetland)^8,9^, and Sulina (Yezo)^10^ nairovirus genogroups, originated in China, Mongolia, and Japan. Another relevant member of the Tamdy genogroup is the Pacific Coast Tick nairovirus (PCTN), isolated from the Pacific Coast tick (*Dermacentor occidentalis*) species which is endemic to the U.S. West Coast and known to transmit other human diseases^11,12^.

The incidence and geographic distribution of CCHFV have expanded in recent decades, driven by the spread of tick vector species into new areas, the occurrence of imported cases, and the emergence of autochthonous transmission^4,13–16^. Such geographical expansion heightens the risk of human-to-human transmission and increases the likelihood of localized outbreaks. In recognition of its growing public health threat, the World Health Organization (WHO) has designated CCHFV as a priority pathogen for the development of improved diagnostics, immunotherapeutic, and vaccines^17,18^. The absence of licensed vaccines or approved therapeutics for prevention or treatment of CCHFV further emphasizes its status as a pathogen with high pandemic potential, emphasizing the urgent need for the development of effective immunotherapeutic strategies^12,19^.

CCHFV is an enveloped virus, with a tri-segmented, negative-sense single-stranded RNA (ssRNA) genome, comprising large (L), medium (M) and small (S) segments^20^. Based on full-genome and segment-specific sequencing, CCHFV strains are currently classified into five major clades: I-III (endemic in Africa), IV (Asia), and V (Europe)^21,22^. During CCHFV infection, low levels of detectable serum antibodies are often correlated with worst disease outcomes, potentially underscoring the supportive role of the humoral immune response in controlling the virus or reducing disease severity^23,24^. Although neutralizing antibodies targeting structural glycoproteins are traditionally considered ideal candidates for vaccines and therapeutic development against other bunyaviruses, in comparison, the utility of neutralizing antibodies against CCHFV is underwhelming^25^. Previous work found that GP38, a glycoprotein and non-neutralizing target, is a key antigen for antibody-based therapeutics^25^. Thus, like those observed with HIV, alphaviruses, flaviviruses, and arenaviruses^25–33^, non-neutralizing antibodies can protect against CCHFV.

The CCHFV nucleocapsid-protein (NP), encoded by the S segment, has emerged as a promising target for non-neutralizing immunotherapeutic development. NP plays a central role in the CCHFV replication cycle, which includes genomic RNA encapsidation, viral ribonucleoprotein (vRNP) complex formation, and host-cell apoptosis regulation^20,34^. It is also the most abundant and highly immunogenic protein in the CCHFV virion, justifying its historical use in CCHFV diagnostics and surveillance assays^20,35–38^. NP-specific IgM is typically the earliest serological marker detected during infection, preceding GPC-specific IgM, and correlating with early control of viral replication^24^. Additionally, recombinant NP (rNP) has been shown to induce humoral responses across sera from various mammalian species^39^. In recent years, a growing number of CCHFV vaccines platforms are incorporating NP as the primary antigen, many of which have been evaluated in animal models^39–44^. Notably, a study using a CCHFV viral replicon particle-based vaccine demonstrated that a non-neutralizing humoral response against NP was a key contributor to rapid protective immunity^39^. Supporting these findings, an NP-targeting monoclonal antibody (mAb) termed 9D5, exhibited broad-spectrum binding affinity to CCHFV strains representing all the five clades, and conferred pre-exposure protection in IFNAR^-/-^ mice challenged with both laboratory-adapted CCHFV IbAr10200 strain (Clade III), and the clinically relevant Afg09-2990 (Afg09) strain (Clade IV)^45^. Collectively, this body of evidence highlights NP as a compelling target for CCHFV immunotherapeutic development.

In this study, four antigenic sites on CCHFV NP were identified using nine identified anti-CCHFV NP antibodies taken from a historic USAMRIID mAb library and the previously reported mAb-9D5^37^. The biochemical and protective nature of these nine antibodies was examined side by side. This included narrowing the location of the four antigenic sites to the stalk, head, or both parts of NP identified through biolayer interferometry (BLI). This revealed that mAb-9D5 binds exclusively to NP head domain, and that mAb targeting other antigenic sites ranged in their ability to provide protection in a murine model. A high-resolution crystal structure of the fragment antigen binding region (Fab) from the most potent mAb, mAb-9D5, bound to CCHFV nucleoprotein originating from CCHFV strain Afg09 was obtained. Crystal structures of unbound NP of CCHFV strain Afg09 and strain Kosova-Hoti (Hoti) were also resolved. These structures identified key amino acids residues critical for mAb-9D5 broad-spectrum protective characteristics. These structural insights combined with the biochemical and efficacy data of the anti-NP mAb paves a path for mAb therapeutic strategies against CCHFV and other bunyaviruses as well as highlights antigens for vaccine development.

## Results

### Murine-derived anti-NP mAb competition and binding kinetics affinity evaluation

**Fig. 1:**
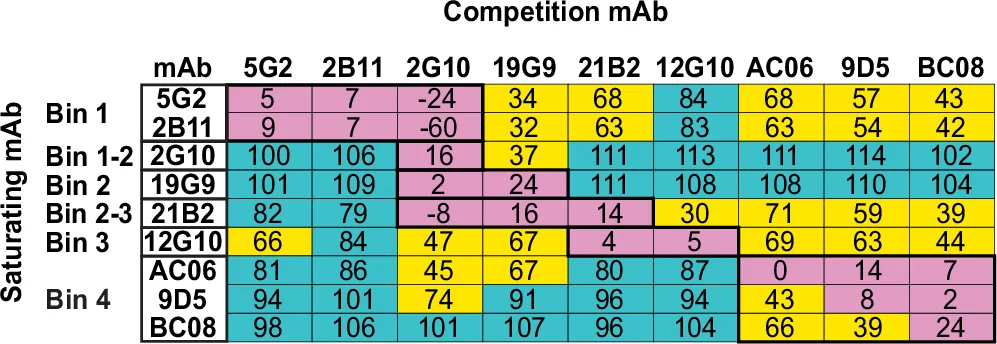
Anti-NP murine non-neutralizing monoclonal antibodies (mAb) competition to CCHFV NP. A panel of nine anti-NP murine mAb was tested to define binding competition to IbAr10200 NP and was classified into four bins (1–4). <30% binding indicates competition (pink), 30–74% indicates partial competition (yellow), and >75% indicates no competition (teal). Data was normalized as percentages by dividing the max raw signal from the competition value by the total signal of Ab2 (competing mAb) binding to sensors without the presence of Ab1 (saturating mAb) and multiplying by 100.

A competition binning and binding kinetics assay was employed to initially characterize a panel of nine different anti-NP murine mAb. In addition to mAb-9D5, this panel contained eight other mAb previously produced at USAMRIID by inoculation of Balb/c mice with CCHFV strain IbAr10200 infected suckling mouse brain homogenates. MAb-AC06 and mAb-BC08, directly competed with mAb-9D5 and were assigned to Bin 4. MAb-5G2 and mAb-2B11 (Bin 1), mAb-19G9 (Bin 2) and mAb-12G10 (Bin 3) showed no or only partial competition with Bin 4 mAb and were each classified into single bins. In contrast, mAb-2G10 and mAb-21B2 exhibited overlapping competition profiles - mAb-2G10 with Bins 1 and 2, and mAb-21B2 with Bins 2 and 3 - and were thus assigned to shared bins, Bins 1–2 and Bins 2–3, respectively (Fig. 1 and Supplementary Fig. 1). Binding kinetics analysis revealed similar dissociation constants (KD) among mAb within the same bin, except for mAb-BC08 (Bin 4) which demonstrated a notably higher KD compared to other Bin 4 mAb (mAb-9D5 and mAb-AC06), indicating a lower binding affinity (Table 1).

**Table 1:** BLI binding kinetics of anti-NP mAb to CCHFV NP IbAr10200

mAb	KD (M)	K*on* (1/Ms)	K*off* (1/s)
**5G2**	2.21 × 10^−10^	8.58 × 10^4^	1.89 × 10^−5^
**2B11**	1.77 × 10^−10^	2.71 × 10^5^	4.80 × 10^−5^
**2G10**	6.31 × 10^−11^	2.13 × 10^5^	1.34 × 10^−5^
**19G9**	9.52 × 10^−11^	1.97 × 10^5^	1.87 × 10^−5^
**21B2**	1.07 × 10^−9^	1.79 × 10^5^	1.92 × 10^−4^
**12G10**	9.84 × 10^−11^	2.17 × 10^5^	2.13 × 10^−5^
**AC06**	4.16 × 10^−10^	1.07 × 10^5^	4.45 × 10^−5^
**9D5**	1.65 × 10^−10^	1.83 × 10^5^	3.01 × 10^−5^
**BC08**	5.62 × 10^−9^	1.61 × 10^4^	8.88 × 10^−5^

### CCHFV anti-NP mAb offered various levels of pre-exposure protection

Recent evidence indicated that humoral immune responses targeting NP are sufficient to confer protection in NP-focused vaccine strategies^39–41,46,47^, with at least one anti-NP mAb (mAb-9D5) demonstrating to mediate protective effects^45^. To identify additional non-competing anti-NP mAb within this panel, that may also confer protection when administered prior to CCHFV exposure, the same mouse model previously used for mAb-9D5 was employed. The potency of these mAb was obtained in two independent experiments using the established IFNAR^-/-^ mouse model (*N* = 8 per group). Mice received intraperitoneal (IP) injections of murine anti-NP mAb (AC06, 12G10, 2B11, 19G9, 5G2, 2G10, BC08, 21B2 and 9D5), or an isotype control antibody on days −1 and +3 relative to challenge. On day 0, all the mice were infected subcutaneously (SC) with 100 plaque-forming-units (PFU) of CCHFV strain Afg09. The mice were monitored daily for clinical signs and group weight loss.

**Fig. 2:**
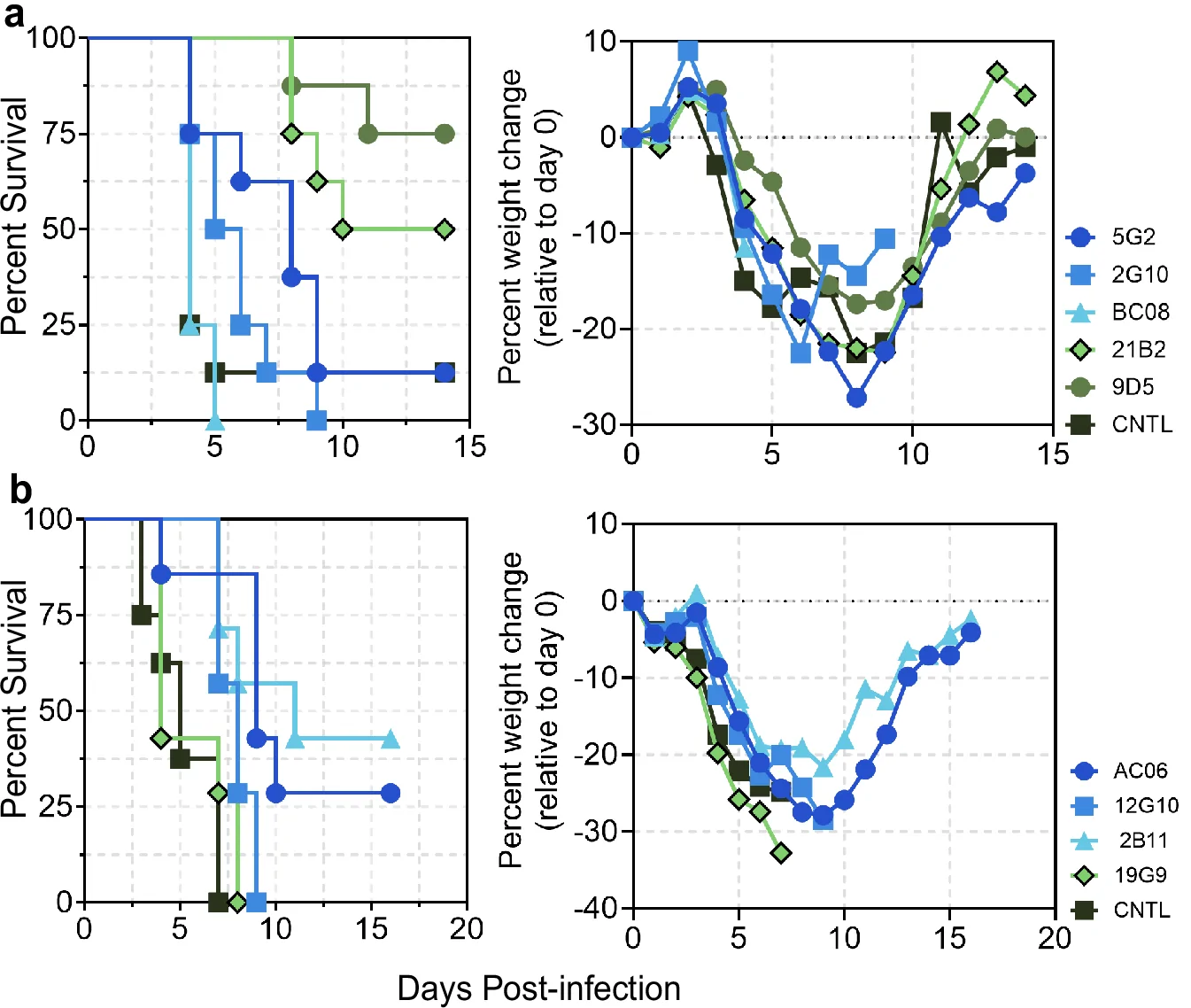
Identification of protective and non-protective mAb targeting CCHFV NP. IFNAR^-/-^ (*N* = 8) mice were treated with two doses (1 mg per 20 g mouse) of anti-NP mAb or an isotype control antibody (CNTL) on days −1/+3 by the IP route. On day 0, all mice were challenged SC with 100 pfu of CCHFV strain Afg09-2990. Percent survival and group weight loss changes were monitored. Weight loss was determined using one-way or two-way ANOVA with the Bonferroni correction. Survival statistics utilized the log-rank (Mantel-Cox) test. Significance levels were set at a *P* value less than 0.05. Anti-NP mAb pre-exposure protection were performed in two independent experiments: **a** Percent of survival and weight change for anti-NP mAb 5G2, 2G10, BC08, 21B2, 9D5 and isotype antibody (CNTL). **b** Percent of survival and weight change for anti-NP mAb AC06, 12G10, 2B11, 19G9 and isotype antibody (CNTL).

The nine antibodies, representing all four competition bins, conveyed varying levels of protection. At the lower end, mice treated with mAb-BC08 (Bin 4) and mAb-2G10 (Bins 1–2) succumbed to infection between days 5 and 9 (Fig. 2a), while mice treated with the isotype antibody, mAb-12G10 (Bin 3) or mAb-19G9 (Bin 2) all succumbed to the infection on days 5, 7, and 8, respectively (Fig. 2b). mAb-5G2 (Bin 1) treated mice showed 12% survival, which was not statistically different from the isotype antibody (Fig. 2a). MAb-AC06 (Bin 4) and mAb-2B11 (Bin 1) fared better in conferring protection, highlighted by a 25% and 40% survival, respectively, with similar weight loss and recovery patterns, followed by mAb-21B2 (Bins 2-3), with 50% survival (*p* = 0.0096), and mAb-9D5 (Bin 4), which is consistent with a recent study^45^, conferred the highest level of protection at 75% survival (*p* = 0.0023). These findings, summarized in Table 2, corroborate previous results, demonstrating that mAb-9D5 significantly enhances survival and delays disease progression in CCHFV-infected mice compared to other anti-NP mAb and the isotype control^45^, highlighting that non-competing anti-NP mAb (2B11 and 21B2) that can significantly protect mice against CCHFV.

**Table 2:** Summary of anti-NP mAb protection of mice. Source data is provided

mAb	Bin	% protection	Delay in MTD	*P* value^a^ vs. control mice
**9D5**	4	75	Y	0.0023
**21B2**	2–3	50	Y	0.0096
**2B11**	1	42.8	Y	0.0017
**AC06**	4	28.6	Y	0.0027
**12G10**	3	0	Y	0.0034
**19G9**	2	0	N	0.3416
**5G2**	1	0	Y	0.1920
**2G10**	1–2	0	N	0.4912
**BC08**	4	0	N	0.6256

*MTD* Median Time to Death, *Y* yes, *N* no.

^a^Log-rank test; two-sided; no adjustments, all comparisons are against the

negative control groups, *P* < 0.05.

### Anti-NP mAb binding preferences to the head or stalk domains of NP

**Fig. 3:**
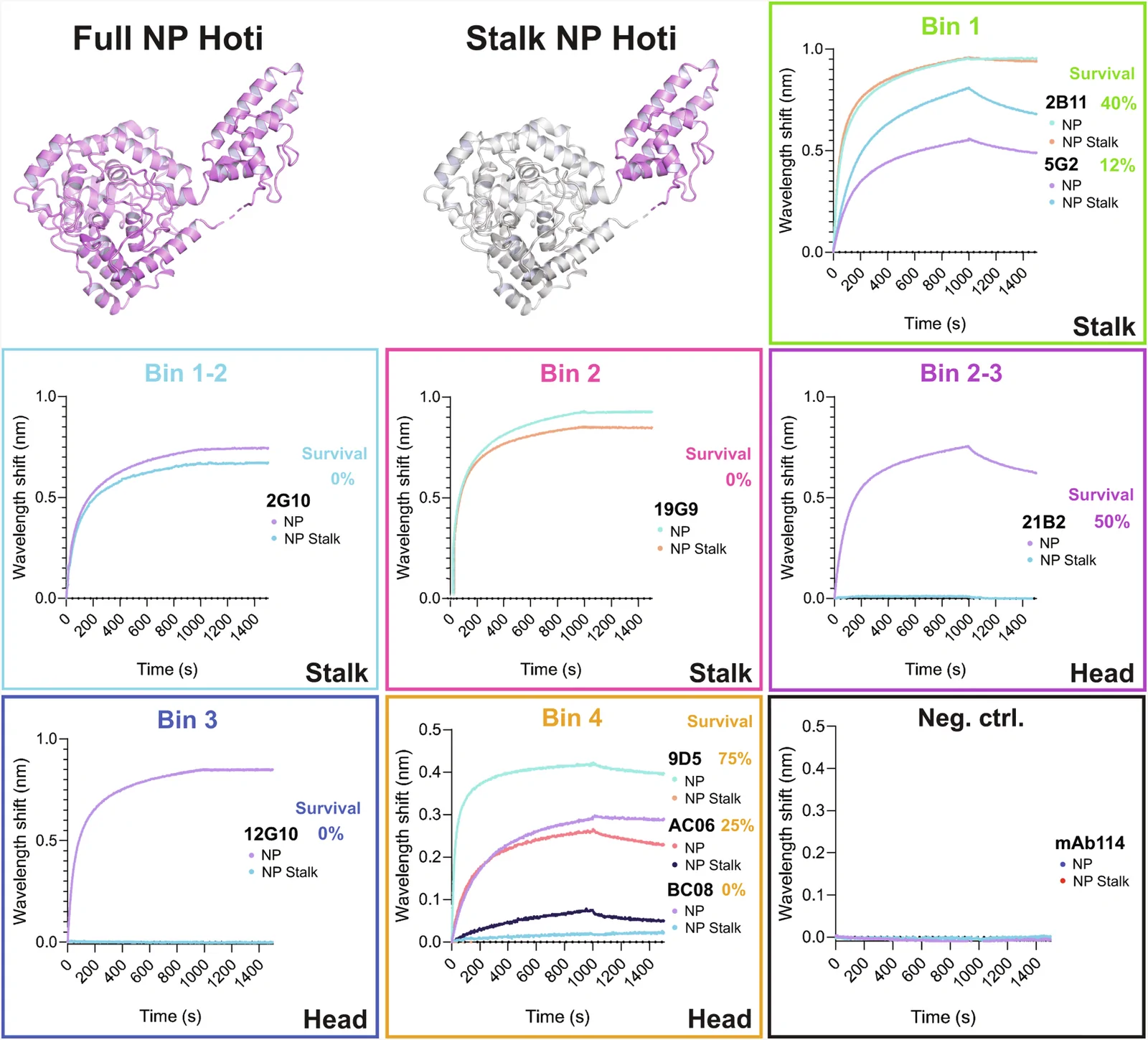
Assignment of Anti-NP mAb epitopes by NP domain. Biolayer Interferometry (BLI) was employed to evaluate anti-NP mAb binding preferences to the full-length NP Hoti strain (10 µg/ml) or NP Hoti stalk domain (30 µg/ml) according to different bins. Top panel: Bin 1 (mAb-5G2 and mAb-2B11), middle panel: Bin 1-2 (mAb-2G10), Bin 2 (mAb-19G9) and Bin 2-3 (mAb-21B2). Bottom panel: Bin 3 (mAb-12G10), Bin 4 (mAb-9D5, mAb-AC06, and mAb-BC08), and mAb114 (negative control) (5 µg/ml).

Previously solved structures of nairovirus nucleoproteins (NPs) have revealed a conserved organization into two primary domains in the monomeric state: the head and the stalk domains^35,48–50^. Notably, the stalk domain exhibits a conformational flexibility, as evidenced by its variable orientations across different nairovirus NP structures^35,49^. To investigate the binding preferences of protective and non-protective anti-NP mAb for the NP head, stalk, or between both domains, biolayer interferometry (BLI) was employed, using either the full-length NP or the isolated stalk domain. Unexpectedly, mAb-9D5 bound exclusively to the full-length NP, indicating it binds to the head domain. This finding contrasts with previous results obtained via liquid chromatography-mass spectrometry (LC-MS) and protease protection which had suggested that the mAb-9D5 epitope was located within the NP stalk region, encompassing amino acid residues 184–208 at the α8^45^. MAb-AC06 and mAb-BC08, also belonging to Bin 4, and mAb-12G10 (Bin 3) revealed the same full-length NP binding preference, with no noticeable wavelength shifts obtained when NP stalk domain was employed. In contrast, mAb assigned to Bin 1 (5G2 and 2B11) and Bin 2 (19G9) demonstrated similar binding capabilities to the full-length NP while also recognized the isolated stalk domain, indicating that their epitopes localize to the stalk or involve interfaces in which the stalk constitutes a major component. Consistently, mab-2G10 (Bins 1-2) and mAb-21B2 (Bins 2-3) displayed binding profiles like those observed for mAb in Bins 1 and 2, and Bins 2 and 3, respectively (Fig. 3). The domain-binding preferences of mab-2G10 and mAb-21B2, together with their bins assignments (Fig. 1), suggest that the observed competition patterns primarily reflect steric hindrance rather than complete epitope overlap. These findings support a refined binning classification, particularly for relatively small antigens where steric effects can confound interpretations of epitope proximity. This phenomenon may also account for the observed unidirectional blocking behavior in the competition assay. Association values (K*on*) for anti-NP mAb binding preferences to full-length or stalk domains NP are shown in (Supplementary Table 1).

### Fine epitope mapping of protective mAb-9D5 to NP Afg09

**Fig. 4:**
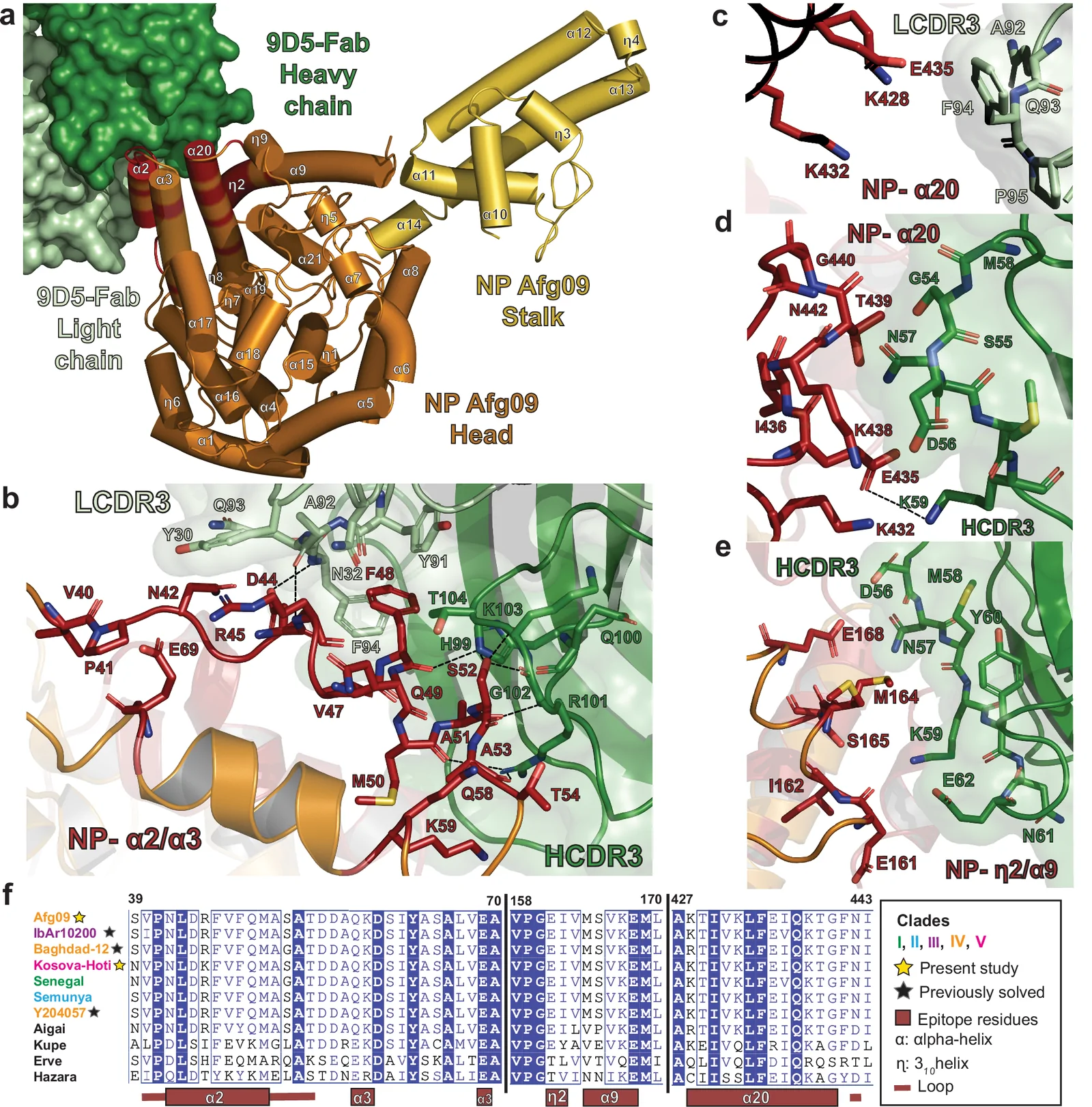
NP Afg09 structure bound to 9D5-Fab. **a** Crystal structure of CCHFV NP Afg09 strain monomer bound to 9D5-Fab heavy-chain (dark green) and light-chain (light green) (PDBID 9ZMQ). NP Afg09 head domain is colored orange and NP Afg09 stalk is colored in yellow. NP alpha-helices (α2, α3, α9 and α20) and 3_10_-helix (η2) highlighted in red represent NP residues interacting with 9D5-Fab epitope area. **b** Magnified NP-α2/α3 residues (red) interactions with 9D5-Fab heavy-chain (HCDR3) and light-chain (LCDR3). **c** Magnified NP-α20 residues (red) detailing interactions with 9D5-Fab light-chain (LCDR3). **d** Magnified detail of NP-α20 residues (red) interactions with 9D5-Fab heavy-chain (HCDR3). **e** Interactions between NP-η2/α9 and 9D5-Fab heavy-chain (HCDR3). Hydrogen bonds are depicted as dash lines for (**a**–**e**). **f** Epitope amino acid comparisons between diverse CCHFV strains and related nairoviruses: Afg09-2990 (accession #HM452305.1 [https://www.ncbi.nlm.nih.gov/nuccore/HM452305.1]), IbAr10200 (accession #MH483987.1 [https://www.ncbi.nlm.nih.gov/nuccore/MH483987.1]), Baghadad-12 (accession #AJ538196.1 [https://www.ncbi.nlm.nih.gov/nuccore/AJ538196.1]), Kosova-Hoti (accession #JN173797.1 [https://www.ncbi.nlm.nih.gov/nuccore/JN173797.1]), Senegal (accession #DQ211640 [https://www.ncbi.nlm.nih.gov/nuccore/DQ211640]), Semunya (accession #DQ076413 [https://www.ncbi.nlm.nih.gov/nuccore/DQ076413]), Y204057 (accession #FJ562093.1 [https://www.ncbi.nlm.nih.gov/nuccore/FJ562093.1]), Aigai virus (accession #NC_078226 [https://www.ncbi.nlm.nih.gov/nuccore/NC_078226]), Kupe virus (accession #NC_078129.1 [https://www.ncbi.nlm.nih.gov/nuccore/NC_078129.1]), Erve virus (accession #JF911699 [https://www.ncbi.nlm.nih.gov/nuccore/JF911699]), and Hazara virus (accession #NC_038711 [https://www.ncbi.nlm.nih.gov/nuccore/NC_038711]). Red bar denotates NP residues interacting with 9D5-Fab.

To better understand the binding area and efficacy of mAb-9D5, a 2.79 Å resolution X-ray crystal structure of 9D5-Fab in complex with NP Afg09 was determined (Supplementary Table 2). The structure was solved in the space group P2_1_2_1_2_1_, with one copy of 9D5-Fab and one copy of NP Afg09 per asymmetric unit. The overall fold of NP Afg09 is consistent with previously solved NP structures, compromising 21 α-helices (α1–α21), and nine 3_10_-helices (η1–η9), organized into a head domain (residues 1–184, 306–481) and stalk domain (residues 194–300)^35,48,49^. The electron density for 9D5-Fab structure and its interface with NP is well-defined, revealing a binding site centered within a highly hydrophobic pocket (Fig. 4a and Supplementary Fig. 3a).

The heavy-chain (HCRD3) and light-chain (LCDR3) regions of 9D5-Fab engage in extensive electrostatic interactions with residues located in four α-helices (α2, α3, α9, and α20) and in one 3_10_-helix (η2) of the NP head domain, spanning both N-terminal (α2, α3, α9 and η2) and C-terminal (α20) regions (Fig. 4a). The core interactions occur in a highly hydrophobic pocket environment NP α2 residues (F48, M50, A51, and A53) and 9D5-Fab light-chain (Y30, Y91, A92, and F94) (Fig. 4b, c). This environment favors the occurrence of several electrostatic interactions between electric charged residues present in the 9D5-Fab heavy-chain pocket (H99, R101, and K103), and 9D5 light-chain (Q93), with NP residues located at α2/α3 (Fig. 4b), α20 (Fig. 4c), and η2/α9 (Fig. 4e). The HCRD3 contributes with a binding interface area of 483.7 Å^2^ and forms numerous hydrogen bonds (H-bonds), particularly with NP α2, α20, and η2/α9 (Fig. 4b, d, e). In contrast, LCRD3 engages fewer residues, primarily in NP α20, and α2/α3, with a smaller interface area of 297 Å^2^ and fewer H-bonds (Fig. 4b, c).

### Structural characteristics influencing mAb-9D5 binding affinities across diverse NP

NP amino acid sequence, and the mAb-9D5 binding epitope area is highly conserved across the five main clades and the CCHFV-like Aigai virus, in contrast to other nairovirus NPs (Fig. 4f and Supplementary Fig. 2). Although mAb-9D5 demonstrated reasonable broad-spectrum binding affinity to multiple CCHFV and Aigai virus NPs, variability in binding strength was observed among different CCHFV strains^45^. To further investigate structural differences in CCHFV NPs that may underline reduced mAb-9D5 binding affinity, we analyzed previously solved NP structures—IbAr10200 (PDBID 4AQG), Baghdad-12 (PDBID 4AKL), and YL04057 (PDBID 3U3I)—and determined two additional X-ray crystal structures for apo-NP Afg09 at 2.54 Å resolution (Supplementary Fig. 3b, d), and apo-NP Hoti at 1.84 Å resolution (Supplementary Fig. 3c, e, and Supplementary Table 2).

**Fig. 5:**
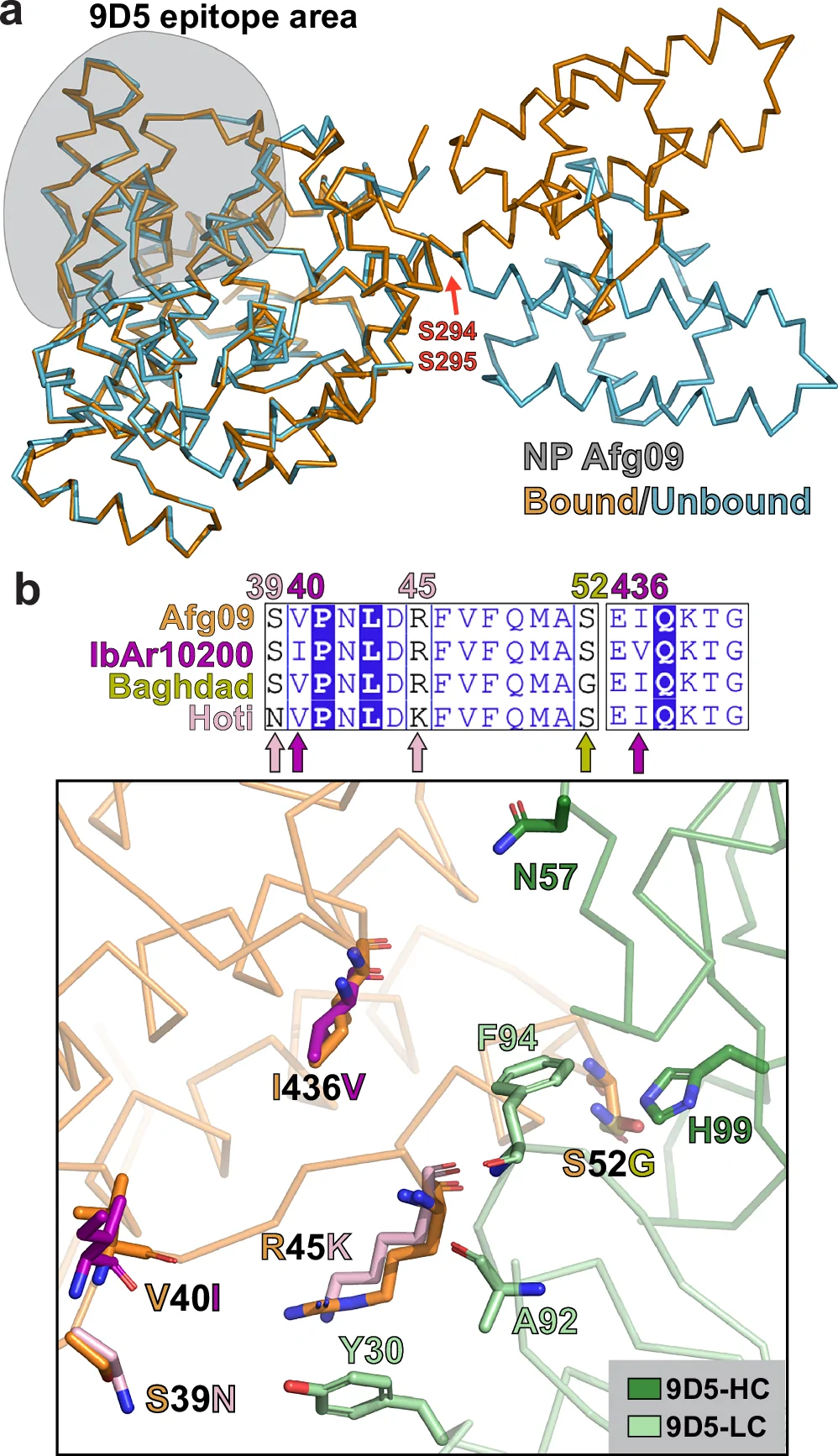
CCHFV NP conserved binding area. **a** Crystal structures superposition of NP Afg09 bound to 9D5-Fab (orange) to NP Afg09 unbound to 9D5-Fab (cyan) highlighting the conserved structural head domain and the flexible conformation of the stalk domain. **b** CCHFV NP binding area residues diversity comparison between known NP structures Afg09 (orange), IbAr10200 (dark pink), Baghdad-12 (limon), and Hoti (light pink).

Superposition of the NP Afg09 structure in complex with 9D5-Fab (bound) onto the apo-NP Afg09 structure (unbound) confirms the previously observed flexibility of the stalk region. A notable angular shift occurs at residues S294–S295, with a calculated Ca root mean square deviation (RMSD) of 0.630 Å across 95 residues. Importantly, the NP 9D5-Fab binding interface retains conserved structural features, as evidenced by a low Ca RMSD of 0.292 Å RMSD across 368 residues within the head domain (Fig. 5a). A similar pattern of conserved or flexible domains was observed in the superposition of additional CCHFV NP structures (Supplementary Fig. 4).

Previously mAb-9D5 association constants values (K_*on*_) determined by BLI were consistent across all tested CCHFV-NP strains^45^. However, NP IbAr10200 (Clade III) and NP Senegal (Clade I) exhibited lower overall binding kinetics (KD) values. In NP IbAr10200, two key amino acid substitutions at the epitope area were identified: an isoleucine in place of a valine at position 40 (I40V), and a valine instead of an isoleucine at position 436 (V436I). These changes may contribute to disrupting the hydrophobic component of the 9D5-Fab interface with NP, leading to increased dissociation rates (K_*off*_) and contributing to the reduced KD value (Fig. 5b). A similar trend was observed for NP Senegal, which displayed comparable K_*off*_ and KD values to NP IbAr10200. Although structural data for NP Senegal are unavailable, it also harbors similar amino acid residue differences, with a valine at position 436 (V436I), as well an asparagine and glycine at positions 39 and 52, respectively, instead of two serine residues (N39S, G52S). NP Hoti (Clade V) also contains two substitutions within the 9D5-Fab binding region. A lysine at position 45 is replacing an arginine (K45R) and may weaken interactions with the 9D5-Fab hydrophobic pocket (LCDR3 residues Y30 and A92). However, the presence of an asparagine at position 39, instead of a serine (N39S), could help stabilize the interaction, resulting in KD values comparable to those of NP Afg09 (Fig. 5b).

## Discussion

Non-neutralizing antibodies (mAb) directed against NP protein have historically received less attention compared to those targeting viral glycoproteins. The efforts to target glycoproteins have seen some success, with mAb targeting GP38, a component of the Pre-G_N_ (M segment) processing in all nairoviruses, showing both pre- and post-exposure protection in murine models^25,29,37,51^. Numerous neutralizing mAb directed against the G_C_ glycoprotein too have been isolated from both infected mice and human survivors with an ability to confer pre-exposure protection^37,52,53^. However, accumulating evidence suggests that NP represents a viable and promising target for combating different viruses, including arenaviruses, influenza, and CCHFV^32,44,45,54–56^. The high abundance and immunogenicity of NP, combined with the lowest genetic diversity among the three segments of CCHFV (approximately 20% across isolates), further supports its appeal as a therapeutic target^3,22^. This is further highlighted by the key role anti-NP humoral responses are demonstrated to play in the durable and potent protection conferred by a recent vaccine candidate^39–41,46,47^ with other vaccines also benefiting from the inclusion of NP^43,44,57^.

Through epitope binning here, at least four distinct binding regions (bins) were identified across the stalk and head domains of NPs. Among these, mAb-9D5 (Bin 4) conferred the highest survival rate in IFNAR^-/-^ mice challenged with CCHFV Afg09 among the nine mAb tested^45^. While AC06, competitive with mAb-9D5, also showed a level of protection which further supports the antigenic site of Bin 4 to be relevant for protection. Bin 4 does not appear to be the only relevant antigenic site within the head region of NP or beyond. The mAb-21B2 that spans antigenic sites Bin 2 and Bin 3 in the head region also confers significant protection whereas mAb spanning Bin 3, or Bin 2 alone does not. In addition to having at least two protective antigenic sites within the head region, mAb-2B11 (Bin 1) demonstrates that significant protection can also occur from a mAb that targets the stalk region. NP stalk domain high antigenicity has been highlighted by other studies performing B-cell epitopes (BCEs) and bioinformatic epitope mapping approaches, and diagnostics^58–61^. Understanding the role of the stalk domain in modulating the protective efficacy of anti-NP mAb targeting this area is crucial for the development of future universal and broadly protective antibodies. The NP stalk domain has also been observed to play a critical role in facilitating conformational changes that enhance both RNA binding affinity and oligomerization^35,49^. While these studies have suggested the importance of the region by the host immune system and to the virus itself, mAb-2B11 suggests that this region can be targeted for mAb therapeutic strategies. These data therefore indicate that multiple domains of NP are viable targeting sites for antibody therapeutics, opening the door to possible synergistic combinations of mAb targeting this molecule. These findings also confirm that different mAb targeting the same antigenic sites (bins) and exhibiting similar binding affinity constants (KD) do not necessarily confer comparable efficacy in vivo^29,62^.

**Fig. 6:**
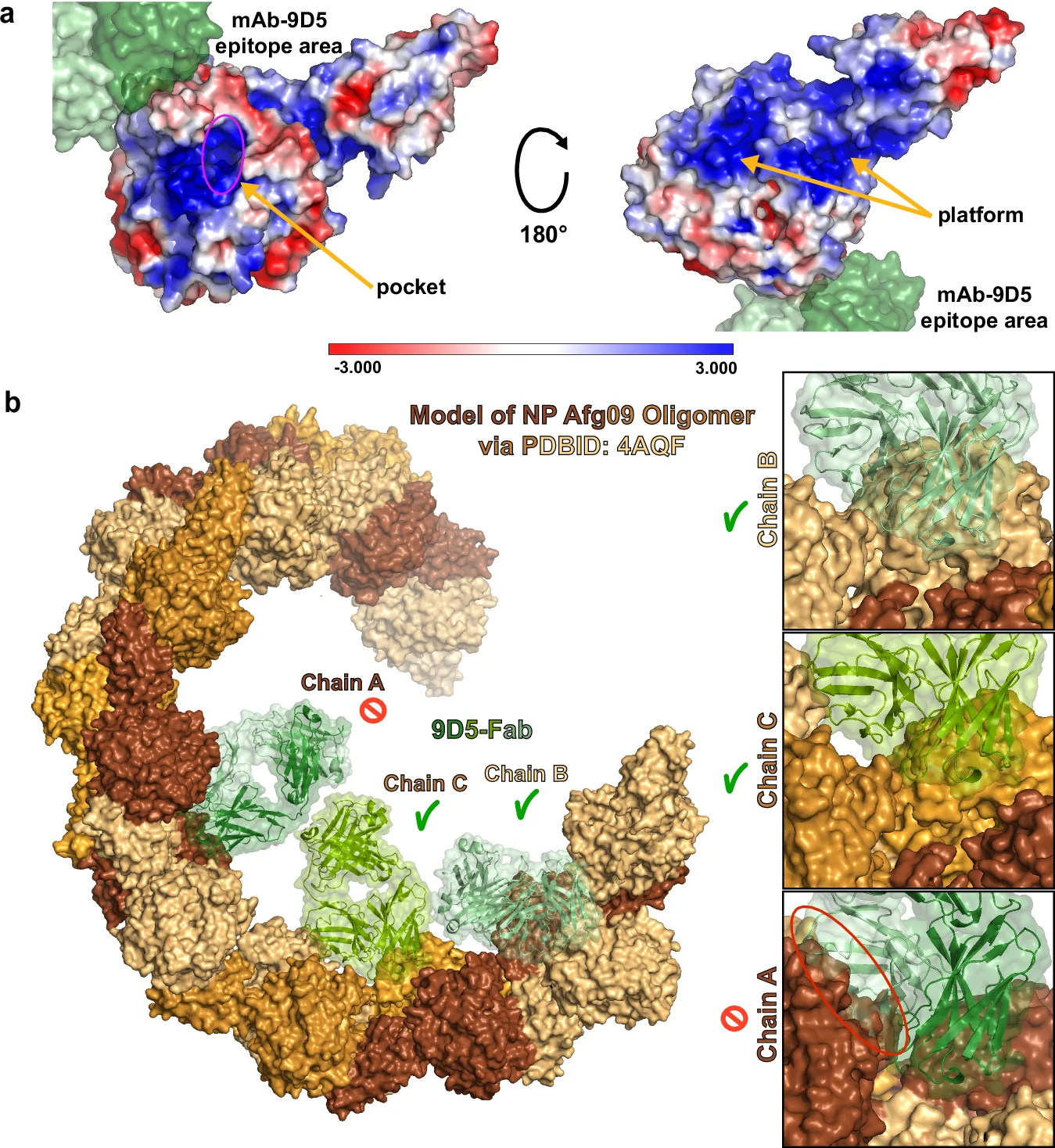
mAb-9D5 epitope accessibility related to surfaces implicated in RNA binding and those previously proposed for oligomerization. **a** NP Afg09 electrostatic surface potential (generated with APBS) indicating positively charged areas (pocket and platform) suggested as RNA binding areas^49^. Pink ellipse indicates area with the residues involved in the NP endonuclease activity; **b** Proposed NP Afg09 oligomer organization based on NP IbAr10200 trimer structure (PDBID 4AQF)^35^ and, mAb-9D5 epitope site accessibility to different NP chains. Red ellipse highlights steric hindrance clash between 9D5-Fab and two protomers of NP Afg09 chain A.

The mAb-9D5-Fab itself offers insight into how anti-NP targeting mAb can take advantage of the conserved nature of antigenic sites located on nairovirus NPs and possible molecular mechanisms of action. The 9D5-NP interface reveals detailed contacts between the hydrophobic pocket of 9D5-Fab and a conserved core of α-helices within the head domain of NP Afg09 (Fig. 4a). Notably, this region exhibits minimal amino acid variation across multiple CCHFV NP strains and is structurally conserved, in contrast to the known NP stalk flexibility. Also, the RNA binding interface is proposed to reside in a highly positively charged region adjacent to the stalk domain and within the head domain pocket^49^. While the affinity for RNA of NP in its monomeric form is considered weak^49^, the RNA binding and endonuclease^50^ regions are spatially distant from the mAb-9D5 epitope area. This indicates whether RNA is bound, that the NP epitope remains accessible for antibody recognition (Fig. 6a). Beyond its monomeric form, NP has been observed to form various oligomeric states during viral replication^63^. NP is detected in early stages of the viral infection and is localized in the perinuclear region of infected cells^20,64^. The NP oligomerization is essential for the encapsidation of newly synthesized viral RNA and the formation of ribonucleoprotein (RNP) complexes^49^. Nucleocapsids of segmented RNA viruses are considered to be more relaxed and irregular shaped compared to those of non-segmented viruses and can be arranged in different morphologies^63^. At the molecular level, insights into NP oligomerization are limited to an X-ray structure and cryo-EM reconstruction that differ in their oligomeric arrangement. The X-ray structure of CCHFV NP strain IbAr10200 (PDBID 4AQF), suggests that RNP adopts an antiparallel head-to-stalk dimeric arrangement, forming a relaxed helicoidal structure with approximately nine NP molecules comprised of three monomeric segments per turn^35^.The cryo-EM reconstruction of CCHFV NP strain YL04057 (PDBID 3U3I) suggests NP multimerization may adopt various ring-shaped conformations, and suggests that CCHFV NP encapsidation may share mechanistic similarities with Lassa virus (LASV) NP^65^. Regarding these models, the mAb-9D5 epitope is exposed. In the helicoidal oligomerization model, two out of three NP molecules per segment (chain B and C) would be accessible. Antibody engagement at sites belonging to chain A is sterically hindered (Fig. 6b). Likewise, mAb-9D5 epitope is also expected to be accessible if the NP adopts a ring-like arrangement observed through Cryo-EM SPA, since this area does not compete with RNA binding during oligomerization. With the absence of a high-ordered CCHFV-NP-RNA structure, neither of these models may be totally reflective of the NPs oligomeric state in the virion. However, the protective nature of mAb-9D5 attests to the possibility of the epitope binding site to be accessible regardless of the NP conformation present.

On the immune system level, the presence of NP on the cell surface during in vitro infection has been established along with its detection in cell-free serum prior to the glycoproteins in some animal models and humans^66–68^. The protective effect of mAb-9D5 has been shown to be independent of complement activation and of surface Fc-receptors^45^. Similar observations have been made for anti-NP antibodies targeting other segmented ssRNA viruses, such as Lymphocytic Choriomeningitis Virus (LCMV)^32^. In contrast, non-neutralizing antibodies against internal proteins of influenza A exhibit distinct mechanisms of action in vitro, including ADCC and antibody-dependent cellular phagocytosis for anti-NP mAb, and an alternative Fc-dependent manner and/or Fc-receptor for matrix protein 1 (M1)^56^. Recent findings have linked the protective mechanism of CCHFV NP-specific antibodies induced by vaccination to the intracellular Fc receptor tripartite motif-containing protein 21 (TRIM21)^44^. TRIM21 is a cytosolic ubiquitin ligase that binds to the Fc regions of antibodies complexed with antigens, targeting them for proteasomal degradation^69^. This mechanism has been implicated in immune responses against both enveloped and non-enveloped viruses, as well as intracellular bacteria^70^. For instance, anti-NP antibodies against LCMV have been shown to promote cytotoxic T-cell responses via TRIM21 and TRIM21 dependent neutralization has been observed for anti-NP IgG and IgM antibodies targeting non-enveloped adenoviruses^71,72^. Non-neutralizing antibodies against internal proteins are readily produced during infection and are usually present in a higher concentration than the neutralizing antibodies targeting surface structural proteins. However, the accessibility of antibody-antigen complexes to TRIM21 in the context of enveloped viruses remains poorly understood. The early detection of high levels of anti-NP antibodies during natural CCHFV infection suggests that NP may be transiently exposed to the cell surface where protective anti-NP mAb like 9D5 can interact with them whether in their monomeric, or oligomeric forms. These complexes could then be internalized into the cytoplasm through a yet unidentified mechanism, where they may be recognized and processed by TRIM21^71^. A future study to address these potential mechanisms is  planned, as the additional mechanistic data will inform the development of both vaccines and therapeutics for CCHF.

In summary, this study characterizes the binding epitope of mAb-9D5, which targets a highly conserved region within the CCHFV NP head domain. This conserved interaction highlights the antibody potential for broad-spectrum applicability against diverse CCHFV strains. Furthermore, several additional protective mAb targeting distinct NP domains, and regions within them, were identified. The identification of protective non-competitive mAb targeting NP at different sites also raises the possibility of enhancing efficacy through use of combinations involving protective mAb targeting NP and other protective antigens. Overall, this work underscores NP targeting antibodies as viable medical countermeasure against CCHFV and emphasizes the utility of these non-neutralizing antibodies in protection.

## Methods

### Ethics statement

All animal study research was conducted under an Institutional Animal Care and Use Committee (IACUC) approved protocol in compliance with the Animal Welfare Act, Public Health Service Policy on Humane Care and Use of Laboratory Animals, and other federal statutes and regulations relating to animals and experiments involving animals. The facility where this research was conducted is accredited by the AAALAC International and adheres to the principles stated in The Guide for the Care and Use of Laboratory Animals, National Research Council, 2011^73^. Animals meeting pre-approved endpoint criteria were humanely euthanized. Female mice were used exclusively as we have not observed sex differences in disease or medical countermeasure evaluation. There are significant limitations for high-containment (BSL4) animal space, therefore we used only one sex to circumvent the need for separate caging. Mice were maintained in an environment with 12 h light/dark cycle, 68–79 °F (set point: 74.5 °F), and 30–70% humidity.

### Anti-NP mAb competition assay and binding kinetics

A Tandem competition assay was used for binning monoclonal antibodies to CCHFV NP recombinant protein. Nickel charged tris-NTA (Ni-NTA) (Pall ForteBio part number 18-5101) sensors were loaded with rNPhis recombinant protein and equilibrated for 10 m in water, then 10 mM Nickel Chloride for 60 s and washed for 60 s in PBS. Sensors were then loaded with 10 µg/ml rNPhis recombinant protein by 5 m incubation in 1× kinetics buffer (ForteBio). Baseline readings were determined by equilibrating sensors for 60 s in 1× kinetics buffer. Purified mAb against NP were diluted with 1× kinetics buffer to a concentration of 100 nM. One column of the Octet sample plate was used for seven mAb and each assay included a no-antibody control well. The eight sensors were incubated in the saturating antibody wells for 5 m before moving the sensors to a baseline well for 60 s in 1× kinetics buffer. The sensors were regenerated by incubating them in solution of 10 mM glycine with a pH of 2.0 for 10 s followed by 10 s in PBS (pH 7.4). This regeneration cycle was performed three times before moving the sensors to a 1 m PBS wash. After washing, sensors were recharged with a 1 m incubation in a 10 mM Nickel Chloride solution. The sensors were then stored in water before being used in additional assays. The data from the sensors was analyzed using the binning function of the Octet analysis software and competition groups assigned. Competition values were normalized as the percent of binding represented by the signal of the competing antibody (Ab2) in the presence of the saturating antibody (Ab1) divided by the signal of Ab2 in the absence of Ab1 on the BLI sensor. Ab1 was loaded on the sensor to fully saturate binding before Ab2 was introduced to the sensor.

Binding kinetics of anti-CCHFV NP mAb were measured on an Octet® Red96e system (Sartorius). Full-length recombinant IbAr10200 strain CCHFV NP expressed in SF9 baculovirus cells, (10 µg/ml) immobilized onto Amine Reactive Second-Generation (AR2G) biosensors (Sartorius) using a standard manufacturer’s amine coupling kit (Sartorius). Following immobilization and quenching, the NP-loaded sensors were dipped into a baseline 1× kinetics buffer (Sartorius) before being exposed to a serial dilution of anti-CCHFV NP mAb, ranging from 1-100 µg/ml, to monitor the association phase over a 600 s period. Subsequently, the sensors were moved into 1× kinetics buffer alone to measure the dissociation phase over a 1200 s period. Data were analyzed using the Octet® Data Analysis HT software (v. 11.1). The sensorgram data were processed by subtracting the reference channel, aligning the Y-axis to the average of the last 10 s of the baseline step, and applying Savitzky-Golay filtering. The processed curves were then globally fitted to a 1:1 binding model to determine the association rate constant (K*on*), dissociation rate constant (K*off*), and the equilibrium dissociation constant (KD).

### Antibody protection studies

IFNAR KO mice (B6.129S2-Ifnar1tm1Agt/Mmjax) were procured from the Mutant Mouse Resource and Research Centers distributed by The Jackson Laboratory. Mice were all female and 7–9 weeks in age at the time of challenge. Mice were challenged with 100 pfu of CCHFV strain Afg09-2990 by the SC route. Virus was diluted in a total volume of 0.2 ml PBS. For antibody injections, mice were injected IP with 1 mg per 20 g mouse (50 mg/kg) dose in a total volume of 0.2 ml diluted in PBS. Weights and survival were monitored and plotted using GraphPad Prism 7 software. Since weight loss is typically not an endpoint criterion for CCHFV, the considered euthanasia criteria employed was lethargy and non-responsiveness.

### mAb binding preference to NP distinct domains

An Octet R8 BLI protein analysis system (Sartorius) was used to measure binding interactions between anti-NP antibodies and His-tagged full-length NP or NP Stalk domain of CCHFV Hoti strain, expressed and purified as described previously^61^ Following initial regeneration step, HIS1K (Anti-Penta-His) sensors (Sartorius) were dipped into baseline 1× Octet kinetics buffer for 60 s. For loading, sensors were dipped into antigen solution for 300 s at 10 µg/ml for full-length NP and 30 µg/ml for NP stalk domain, followed by a second baseline step. Sensors were then dipped into 5 µg/ml of monoclonal antibody solutions for 1000 s as an association step and then transferred back to 1× Octet kinetics buffer for 500 s for the dissociation step. Binding experiments were performed in duplicate, and binding responses were recorded as wavelength shift in nanometers (nm). Data were reference-subtracted using buffer only control sensors. An irrelevant monoclonal antibody, mAb114, served as a negative control. The analysis performed using Octet data analysis software version 12.2 and sensongrams were plotted using Graphpad Prism 10.

### Expression and purification of CCHFV NPs and anti-NP 9D5-Fab

Plasmid constructs pET-28a (+) expressing full-length NP Afg09 and NP Kosova-Hoti were expressed and purified as previously described^45^. 9D5-Fab were obtained by transfecting Expi293 cells (Thermo Fisher, Cat. # A14527) with a 1:1 ratio of His-tagged heavy-chain to untagged light-chain plasmid DNA (pTwist) (Twist Technologies) using FectoPro reagent (Polyplus). Fab containing supernatant was harvested between 5 and 6 days, and the cell debris was removed by centrifugation and filtered through a 0.2 μm filter membrane. The supernatant was equilibrated and purified by immobilized metal chromatography using HisTrap-Excel nickel column (Cytiva). 9D5-Fab fractions were eluted using 500 mM imidazole, combined, and purified by size exclusion chromatography on a Superdex 75 column. NPs and 9D5-Fab final products were confirmed by SDS-PAGE electrophoresis (BioRad) and concentrated to a final concentration of 10–12 mg/ml using a 30 K and a 10 K VivaSpin Centrifugal Concentrator, respectively (Sartorius).

### Crystallization of NP Afg09 9D5-Fab complex, NP Afg09, and NP Hoti

NP Afg09 9D5-Fab complex was obtained by combining a ratio of 1:1.2 M, respectively. The proteins were equilibrated to 4 mM Tris-HCl pH 7.4 and 200 mM NaCl, and incubated up to 16 h at 4 °C. The complex was purified over a Superdex 200 column (Cytiva), and the fractions were collected and run using SDS-PAGE electrophoresis to confirm purity. Fractions containing the complex were then concentrated to 10–12 mg/ml using a 30 K Vivaspin Centrifugal Concentrator. Nextal crystal screen solutions were plated via an SPT Lab Tech Mosquito using a 1:1 ratio of protein to well solution hanging drop to a total volume of 600 nl. Drops were checked over 2–3 weeks for crystal formation. NP Afg09 9D5-Fab crystals were formed in a solution of 0.1 M Tris pH 8.0, 0.2 M KCl, 6% PEG 6000, 3% w/v Trimethylamine N-oxide dihydrate as additive (Hampton Research). The crystal was flash-frozen in liquid nitrogen using PEG 8000 30% as cryopreserving. Apo-NP Afg09 crystals were formed in a solution of 0.8 M MgCl_2_, 19% PEG 3350, 0.1 M HEPES, 0.05 M sodium fluoride and flash-frozen in liquid nitrogen using PEG 3350 30% as cryopreserving. Apo-NP Hoti crystals were formed in a solution of 1 M MgCl_2_, 0.1 M Bis-Tris, 25% PEG 3350, 1% v/v 1,2-Butanediol as additive (Hampton Research) flash-frozen in liquid nitrogen in mother liquor cryopreserving.

### Data collection, processing, and refinement

NP Afg09 9D5-Fab was collected at Brookhaven National Laboratory (BNL), National Synchrotron Light Source II, beamline FMX (17-ID-2). Data for apo-NP Afg09 and apo-NP Hoti were collected at Stanford Synchrotron Radiation Lightsource (SSRL), SLAC National Accelerator Laboratory, beamline BL9-2. Data Processing was performed using HKL-2000 (v 719.2) and CCP4 suite (v 0.9.8.91 EL). To phase the complex NP Afg09 and 9D5-Fab models were generated using MODELLER (v 10.4) (PDBID 4AQG, and 8DCY, respectively) and Molecular Replacement was performed using Phaser-MR (full-featured). Apo-NP Afg09 and apo-NP Hoti models were generated employing only the NP Afg09 from the complex structure previously solved in this study, followed by Phaser-MR (simple one-component interface) out of the Phenix suite of programs. After phasing, structures went through multiple rounds of refinement in Coot (v 0.9.8.3) and Phenix (v1.21.rc1).

### Statistical analysis

Weight loss was determined using one-way or two-way ANOVA with the Bonferroni correction. Survival statistics utilized the log-rank (Mantel-Cox) test. Significance levels were set at a *P* value less than 0.05. All analyses were performed using GraphPad Prism 7 software.

## Supplementary information

Supplementary Information

Transparent Peer Review file

## Source data

Source Data

## Data Availability

All data generated or analyzed during this study are included in this published paper, source data file, and supplementary. Data that support the findings of this study are also available from the corresponding author upon request. Atomic coordinates and structure factors have been deposited in the Protein Data Bank with PBDIDs 9ZMQ, 9ZMR, and 9ZMS. The data used in this study are available in the Protein Data Bank database under accession code 4AQG, 4AQF, 4AKL, 8DCY, and 3U3I. The following existing NP protein sequences utilized in this study were obtained from NCBI GenBank: Afg09-2990 (accession #HM452305.1 [https://www.ncbi.nlm.nih.gov/nuccore/HM452305.1]), IbAr10200 (accession #MH483987.1 [https://www.ncbi.nlm.nih.gov/nuccore/MH483987.1]), Baghadad-12 (accession #AJ538196.1 [https://www.ncbi.nlm.nih.gov/nuccore/AJ538196.1]), Kosova-Hoti (accession #JN173797.1 [https://www.ncbi.nlm.nih.gov/nuccore/JN173797.1]), Senegal (accession #DQ211640 [https://www.ncbi.nlm.nih.gov/nuccore/DQ211640]), Semunya (accession #DQ076413 [https://www.ncbi.nlm.nih.gov/nuccore/DQ076413]), Y204057 (accession #FJ562093.1 [https://www.ncbi.nlm.nih.gov/nuccore/FJ562093.1]), Aigai virus (accession #NC_078226 [https://www.ncbi.nlm.nih.gov/nuccore/NC_078226]), Kupe virus (accession #NC_078129.1 [https://www.ncbi.nlm.nih.gov/nuccore/NC_078129.1]), Erve virus (accession #JF911699 [https://www.ncbi.nlm.nih.gov/nuccore/JF911699]), and Hazara virus (accession #NC_038711 [https://www.ncbi.nlm.nih.gov/nuccore/NC_038711]). Source data are provided as a Source Data file. Source data are provided with this paper.
