# Supplementary material for: Structural and mechanistic insights into protective non-neutralizing antibodies targeting Crimean-Congo hemorrhagic fever virus nucleocapsid protein
Source: Nat Commun. 2026 Aug 21;17:9967. doi: 10.1038/s41467-026-76702-1 (PMC13586249; doi:10.1038/s41467-026-76702-1)
Supplement: Supplementary file 1 [file 41467_2026_76702_MOESM1_ESM.pdf]

Structural and mechanistic insights into protective non-neutralizing antibodies targeting Crimean-Congo hemorrhagic fever virus nucleocapsid protein

Vanessa Moresco[1‡], Aura R. Garrison[2‡], Clarissa A. Edmundo[1], Collin J. Fitzpatrick[2,3], Elif Karaaslan[4], Scott P. Olschner[5], Keersten M. Ricks[5], Oluwadara T. Ogundare[1], Laliv Tadri[1], Brian D. Carey[2,3], Mohammad M. Sajadi[6], Éric Bergeron[4], Joseph W. Golden[2*] and Scott D. Pegan[1,7*]

[1]*Division of Biomedical Sciences, University of California Riverside, Riverside, CA, USA.*

[2]*Virology Division, United States Army Medical Research Institute of Infectious Diseases, Fort Detrick, MD 21702.*

[3]*Chenega Professional and Technical Service.*

[4]*Viral Special Pathogens Branch, Division of High-Consequence Pathogens and Pathology, Centers for Disease Control and Prevention, Atlanta, GA, USA.*

[5]*Diagnostic Systems Division, United States Army Medical Research Institute of Infectious Diseases, Fort Detrick, MD 21702.*

[6]*Institute of Human Virology, University of Maryland School of Medicine, USA.*

[7] *Department of Chemistry and Biological Science, and Engineering, United States Military Academy, West Point, NY, USA.*

[‡]These authors contributed equally.

[*]Corresponding authors: joseph.w.golden.civ@health.mil and scott.pegan@medsch.ucr.edu

SUPPLEMENTARY FIGURES AND TABLES

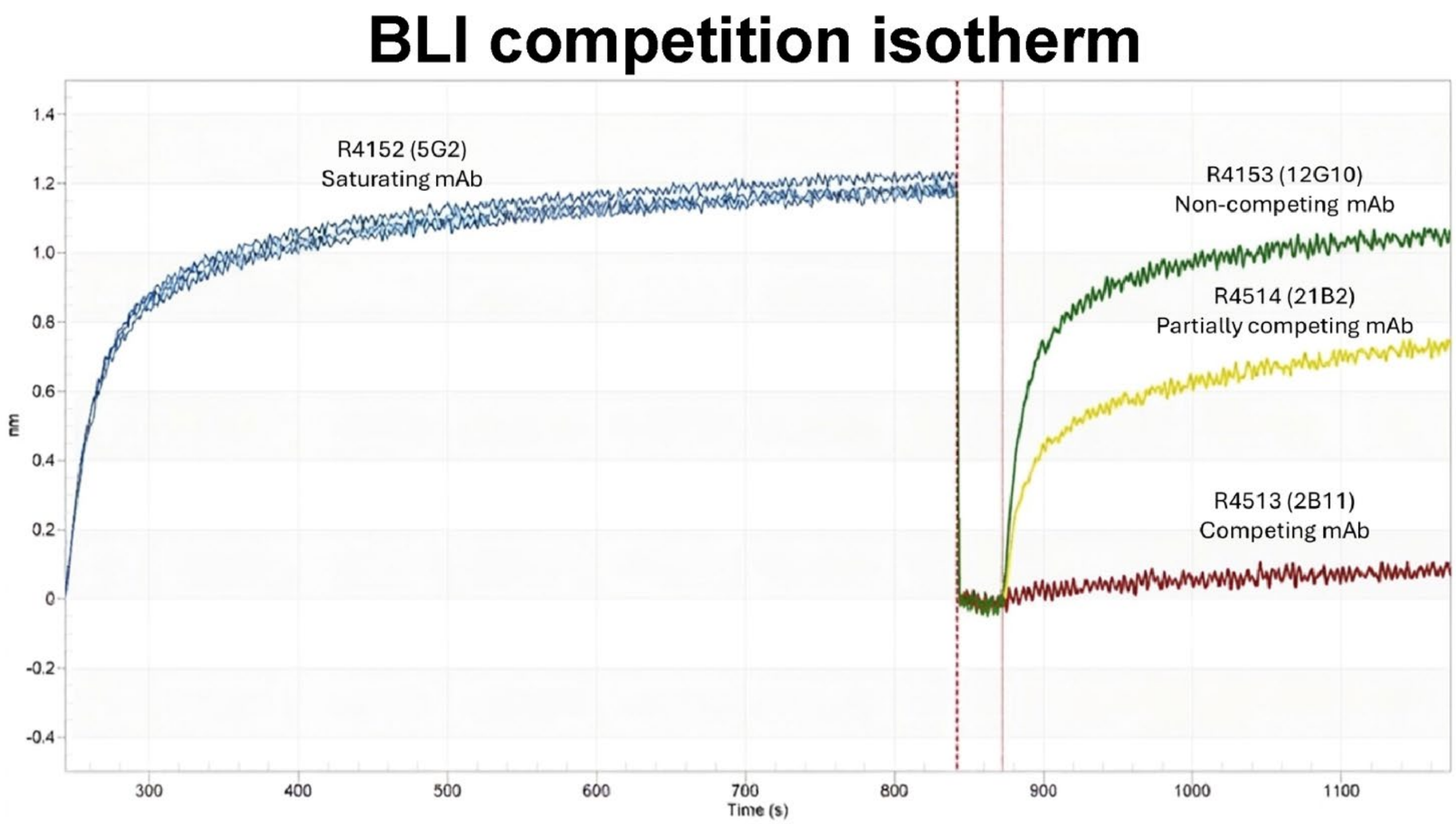

Supplementary Fig. 1. Saturating mAb-5G2 representative raw isotherm curves showing non-competing (mAb-12G10), partially competing (mAb-21B2), and competing (mAb-2B11) binding. All mAb were introduced to the sensors at 100 μg/ml. The graph shows the saturation step followed by an equilibrium/baselining step, followed by a competition step.

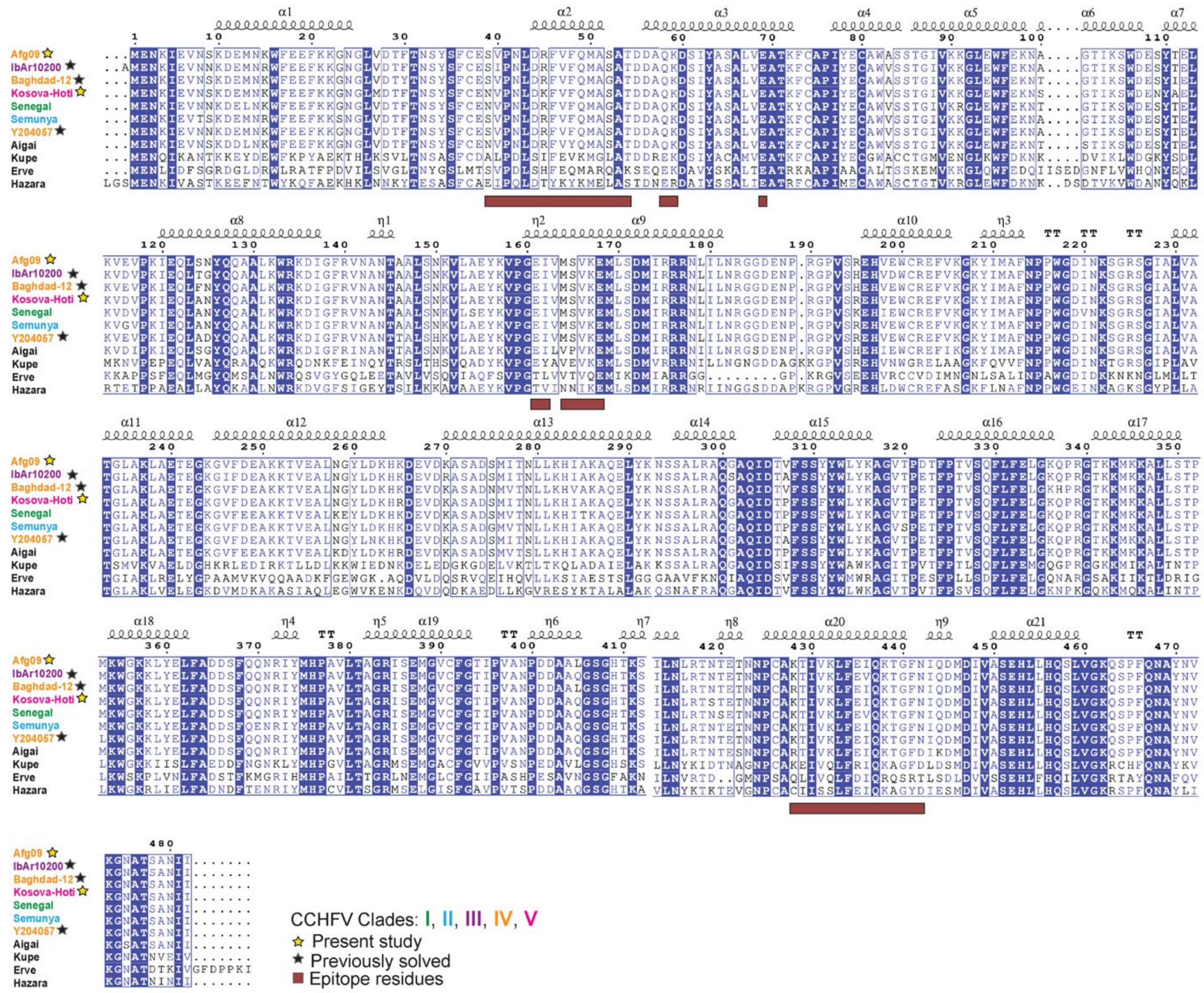

Supplementary Fig. 2. Sequence alignment of full-length NP CCHFV strains sequences representing the five different clades and related nairoviruses. Afg09-2990 (accession #HM452305.1), IbAr10200 (accession #MH483987.1), Baghadad-12 (accession #AJ538196.1), Kosova-Hoti (accession #JN173797.1), Senegal (accession #DQ211640), Semunya (accession #DQ076413), Y204057 (accession #FJ562093.1), Aigai virus (accession #NC_078226), Kupe virus (accession #NC_078129.1), Erve virus (accession #JF911699), and Hazara virus (accession #NC_038711). Similarity and alignment calculations were performed using Clustal Omega. General outline and graphic were generated using EsPript. Residue positions of high conservation are highlighted in blue.

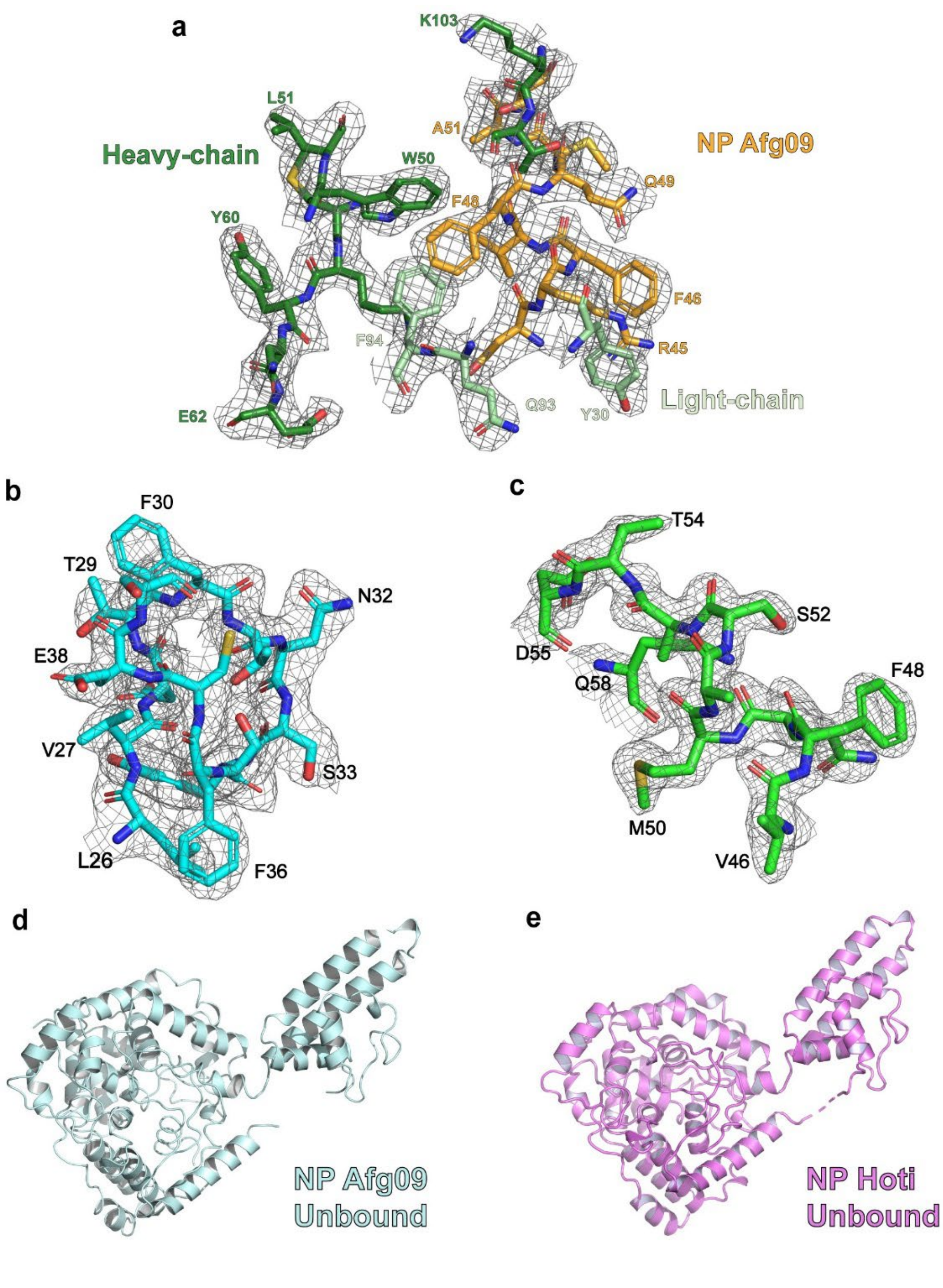

Supplementary Fig. 3. Electron density maps of the new crystallographic structures. a. NP Afg09 bound to 9D5-Fab (PDBID 9ZMQ); **b.** NP Afg09-apo (PDBID 9ZMR); **c.** NP Hoti-apo (PDBID 9ZMS). Electron density map ($2F_o$ - F_c) is presented as a grey mesh contoured at 1.0 σ; **d.** Crystal structure of NP Afg09-apo (PDBID 9ZMR); **e.** Crystal structure of NP Hoti-apo (PDBID 9ZMS).

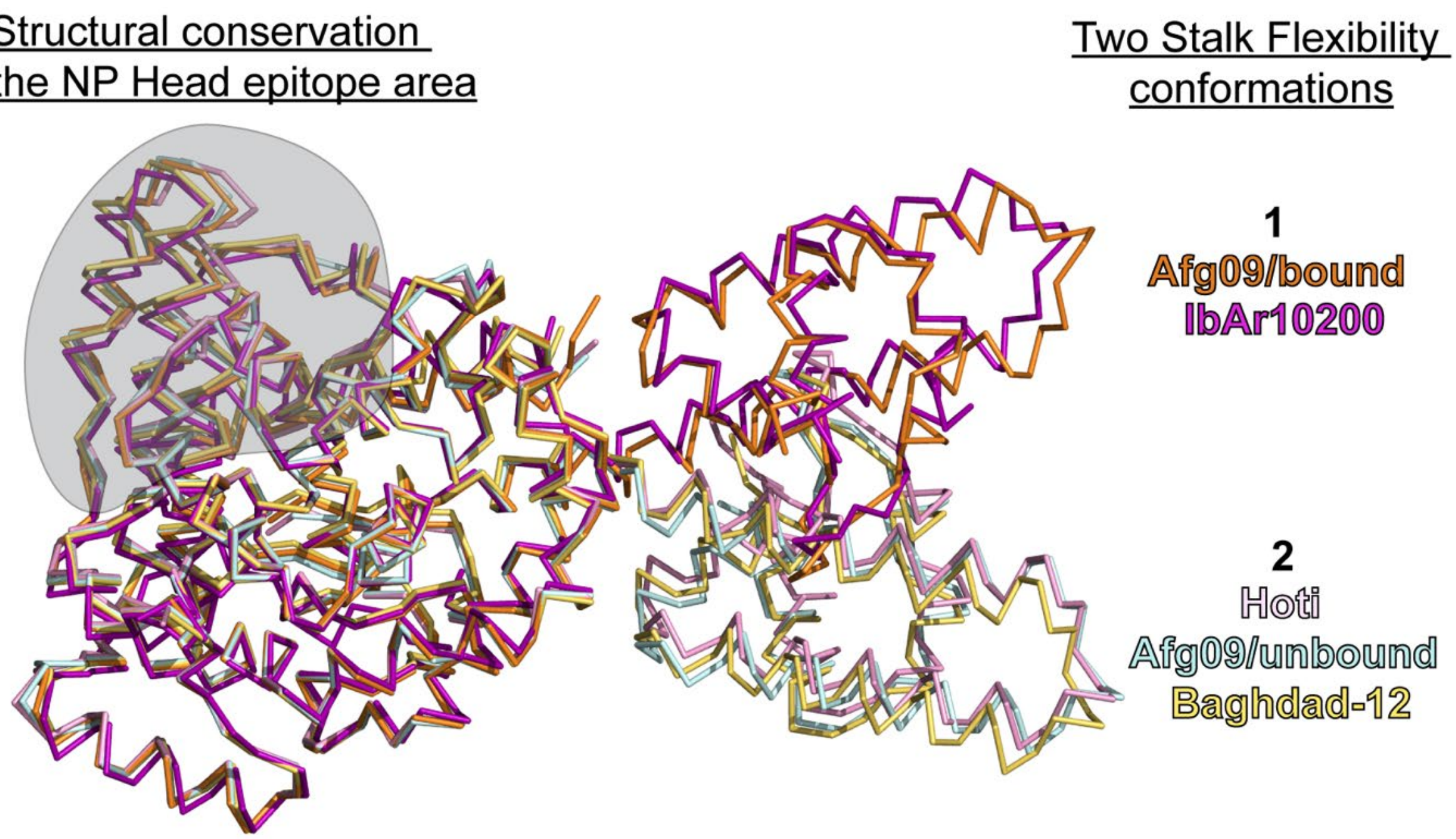

Supplementary Fig. 4. NP head domain structural conservation and stalk domain flexibility conformations across CCHFV strains. Overlay of NP Afg09 bound to 9D5-Fab (PDBID 9ZMQ), apo-NP Afg09 (PDBID 9ZMR), apo-NP Hoti (PDBID 9ZMS), IbAr10200 (PDBID 4AQG), and NP Baghdad-12 (PDBID 4AKL).

Supplementary Table 1. Association constant (K*on*) of anti-NP mAb binding preferences to NP Hoti full-length or stalk domains.

	Full-length NP	Stalk NP
**mAb**	**K*on* (1/Ms)**	**K*on* (1/Ms)**
**5G2**	1.32×10^5	1.12×10^5
**2B11**	2.76×10^5	3.08×10^5
**2G10**	2.32×10^5	2.29×10^5
**19G9**	2.9×10^5	3.26×10^5
**21B2**	2.13×10^5	NA
**12G10**	2.92×10^5	NA
**AC06**	1.59×10^5	NA
**9D5**	6.84×10^5	NA
**BC08**	1.22×10^5	NA

NA: no association

Supplementary Table 2. Data collection and refinement statistics.

	NP Afg09	NP Hoti	NP Afg09 complex with 9D5-Fab
**PDB**	**9ZMR**	**9ZMS**	**9ZMQ**
**Data collection**			
Space group	$P2_12_12_1$	$P2_12_12_1$	$P2_12_12_1$
Wavelength (Å)	0.097	0.097	0.9795
Cell dimensions			
a, b, c (Å)	73.745 86.555 155.993	72.794 85.762 154.332	85.304 94.98 121.839
a, b, g (°)	90.00 90.00 90.00	90.00 90.00 90.00	90.00 90.00 90.00
Resolution (Å)	50.00-2.54 (2.54 – 2.5)	50.00-1.87 (1.87-1.84)	50.00-2.79 (2.85-2.8)
R_{pim} (%)	4.3 (31.4)	4.2 (68.5)	12.3 (30.6)
R_{meas} (%)	8.3 (61.9)	11.0 (174.4)	24.4 (59.8)
$cc_{1/2}$	0.992 (0.787)	0.996 (0.416)	0.943(0.705)
I/s (I)	19.38 (1.94)	19.98 (0.78)	4.6 (1.28)
Completeness (%)	99.70 (100)	99.2 (89.8)	99.8 (97.3)
Redundancy	6.8 (7.2)	6.7 (6.29)	7.3 (6.8)
**Refinement**			
Resolution (Å)	44.58 - 2.503 (2.58 – 2.5)	44.12 – 1.844 (1.89 – 1.84)	38.91 - 2.79 (2.91 – 2.79)
No. of reflections	34982 (2795)	83478 (5691)	24517 (2620)
R_{work} (%)/R_{free} (%)	21.36/25.42	18.15/19.17	17.45/22.61
No. atoms			
Protein	7310	7438	7022
Water	125	557	305
B-factors			
Protein	72.27	38.54	20.99
Water	47.17	38.20	18.86
RMSD			
Bond lengths (Å)	0.003	0.015	0.002
Bond angles (%)	0.50	1.18	0.46
Estimate of coordinate error	0.25	0.24	0.25

*Highest resolution shell is shown in parenthesis.
