# Supplementary material for: Structural and mechanistic insights into protective non-neutralizing antibodies targeting Crimean-Congo hemorrhagic fever virus nucleocapsid protein
Source: Nat Commun. 2026 Aug 21;17:9967. doi: 10.1038/s41467-026-76702-1 (PMC13586249; doi:10.1038/s41467-026-76702-1)
Supplement: Supplementary file 2 [file 41467_2026_76702_MOESM2_ESM.pdf]

nature portfolio

Peer Review File

Structural and mechanistic insights into protective non-neutralizing antibodies targeting Crimean-Congo hemorrhagic fever virus nucleocapsid protein

Corresponding Author: Dr Scott Pegan

This file contains all reviewer reports in order by version, followed by all author rebuttals in order by version.

Version 0:

Reviewer comments:

Reviewer #1

(Remarks to the Author)
In the present manuscript, Moresco, Garrison and colleagues follow up on their previous characterization of anti-CCHFV (Crimean Congo hemorrhagic fever virus) nucleocapsid monoclonal antibodies (mAbs) PMID: 38409240. The current manuscript reports additional mAbs developed at USAMRIID. The authors bin the mAbs to epitopic groups, perform mouse protection studies and report a crystal structure of the 9D5 Fab bound with CCHFV nucleocapsid protein.
Overall, the manuscript offers a first study that correlates epitopes on NP with in vivo protection. It is a relatively straightforward study, but several issues need to be addressed.

Major:

1 – The antibody dose in mouse protection studies is massive. The authors report 1mg/mouse/dose with is approximately 40 mg/kg. Typically, mouse protection experiments do not exceed 20 mg/kg. In the absence of new mouse experiments, the authors should at least clearly state the dose in mg/kg in both the figure legend and the text and discuss the relevance of such a high dose.

2 – The model of mAb 9D5 binding to the NP oligomer is highly speculative. The authors should perform negative stain EM experiments to show that. Physiologically, a bivalent IgG molecule, as opposed to Fab, would likely bind and therefore disrupt the NP oligomer. This should be experimentally tested and discussed in the text.

Minor:

1 – The BLI competition isotherms should be reported in the supplementary data

Reviewer #2

(Remarks to the Author)
This manuscript follows the authors' prior work on the monoclonal antibody 9D5 against Crimean-Congo hemorrhagic fever virus nucleoprotein (CCHFV NP). In this study, they attempt to identify more potent anti-NP monoclonal antibodies (mAbs) and classify them into groups using competition assays. They also resolve the complex structure of NP bound to 9D5. While the study addresses an important objective, the results are limited: no mAbs more potent than 9D5 were identified. Moreover, 9D5 itself exhibits only modest in vivo efficacy (~50%-75% protection when administered prophylactically, based on their previous and current studies). The binning analysis does not establish a clear correlation between in vivo protection and binding epitopes, which impairs the overall significance of the work.

Since structural biology is not my area of expertise, my comments are limited to the biological aspects:

Figure 1—Binning of anti-NP monoclonal antibodies
1.Four colors are used in Figure 1a, but the legend describes only three: pink (≥30% binding inhibition, competition), yellow

(30–75%, partial competition), and green (>74%, no competition). What does the cyan color represent?

Additionally, should the thresholds be defined as 30–74% for partial competition (yellow) and ≥75% for no competition (green)?

It is also unclear how the competition values were defined/normalized.

2.Many competition interactions appear unidirectional, which complicates interpretation. For example:
A. mAb 12G10 (Bin 3–4) shows weak competition (69%/63%/44%) with three mAbs in Bin 4, but no competition is observed in the reverse direction.
B. Similarly, Bin 2–3 shows only one-directional competition with Bin 2.
C. mAb 2G10 (Bin 2) competes strongly with two mAbs from Bin 1 in one direction.

These inconsistencies make the data difficult to interpret and weaken support for the proposed grouping.

Figure 2—In vivo protective efficacy

1.Although final survival rates for 9D5 and 21B2 were 75% and 50%, respectively, both groups experienced severe weight loss (~20%), indicating only modest protection. None of the newly identified mAbs outperformed 9D5.

2.The IFNAR-/- mouse model is semi-self-limiting for CCHFV infection; survivors after 7 dpi typically clear the virus and regain weight. The ethical endpoint criterion is not specified. If weight loss >30% was used, a more stringent threshold (e.g., >20%) would likely further reduce reported survival rates.

3.Given that survival outcomes depend on the ethical endpoint criteria, additional measures of protection should be provided. These should include viral loads (in liver, spleen, and serum) and histopathological analysis (e.g., H&E staining) of affected tissues.

Figure 3—Assignment of Anti-NP mAb epitopes by NP domain

1.Please provide a table listing the KD values for each mAb–NP pair. The binding curve for Bin 4 appears unusual and warrants explanation.

2.I don’t understand why mAbs within the same bin (e.g., Bin 4: 9D5 and AC06, which show similar KD values for Afg09 NP in Figure 1b) exhibit different in vivo protective efficacy.

Overall Recommendation
The study presents valuable structural data and a systematic binning analysis. However, the failure to identify superior mAbs and the lack of clear correlation between epitope groups and in vivo protection limit the translational significance of the findings. Including additional virological and pathological data, as noted above, would strengthen the manuscript.

Reviewer #3

(Remarks to the Author)
In this manuscript, Vanessa Moresco et al. identify nine monoclonal antibodies targeting CCHFV NP and systematically characterize their biochemical properties and in vivo protective efficacy. Four epitope bins are defined, among which mAb-9D5, assigned to bin4, confers approximately 75% protection. The authors further determine the structure of the NP-9D5 complex, delineate the precise binding epitope, and, together with sequence analysis, propose a molecular basis for its potential broad protective activity. These findings offer meaningful insights into the functional complexity of CCHFV NP and provide a valuable foundation for developing antibody-based therapeutics and vaccines. To further strengthen the impact and rigor of this already substantial work, a few aspects could benefit from additional elaboration and contextual discussion.
Major concerns:
1.For Figure 6 and the related discussion, I suggest the authors reorganize their analysis in the context of the current CCHFV RNP structural models. As these existing models are derived from recombinantly expressed proteins (whether solved by X-ray crystallography or EM), the proposed RNP architecture may not represent the authentic viral complex. Consequently, the structural superposition of the NP-Fab complex onto the NP oligomer model carries limited biological significance.
2.Line183-188, For epitope mapping, the authors compared antibody binding to the NP stalk region and to the full-length NP protein, and antibodies that bound only to the full-length NP were inferred to recognize the head domain. Could the authors clarify why the head domain itself was not directly tested? Direct binding assays using the isolated head domain would strengthen the conclusions and provide a more straightforward interpretation. A relevant construct design can be found in this literature: Structural and Functional Diversity of Nairovirus-Encoded Nucleoproteins./jvi.01680-15
3.Among the three antibodies classified into bin4, only 9D5 exhibits protective activity. Given that the other two antibodies compete for the same epitope site, the authors are suggested to explain why these antibodies were not further characterized structurally, as such analyses could provide additional insight into the relationship between this epitope cluster and protective efficacy.
Minor issue:
1.Line 181-183, while the cited literature covers important aspects of CCHFV NP structures, the background would benefit from including the pioneering work that resolved the first monomeric NP structure and characterized its endonuclease

function. Crimean-Congo hemorrhagic fever virus nucleoprotein reveals endonuclease activity in bunyaviruses, Proc. Natl. Acad. Sci. U.S.A., vol. 109, no. 13, pp. 5046–5051, 2012. doi: 10.1073/pnas.1200808109.
2.In Figure 3, the BLI traces appear relatively thick, making it difficult to visualize the binding curve of 9D5 to the NP stalk within bin4. Adjusting the line thickness would improve clarity.
3.In Figures 4b-d, hydrogen bonds should be explicitly labeled to allow clear distinction from hydrophobic interactions.
4.The order of the subpanels in Figure 4 should match their order of appearance in the main text. In addition, the figure legends use capital letters whereas the in-text and figure labels are lowercase; please ensure consistency with the journal's formatting requirements.
5.Lines 226-227, Please note that the manuscript uses both en dashes (–) and em dashes (—) inconsistently. The authors are suggested to ensure consistency throughout and use the correct dash for each function.

Version 2:

Reviewer comments:

Reviewer #1

(Remarks to the Author)
No further comments

Reviewer #2

(Remarks to the Author)
The authors have addressed my previous concerns.

Reviewer #3

(Remarks to the Author)
The authors have thoroughly addressed all my previous concerns in this revision. I have no further comments and recommend the manuscript for publication.

Open Access This Peer Review File is licensed under a Creative Commons Attribution 4.0 International License, which permits use, sharing, adaptation, distribution and reproduction in any medium or format, as long as you give appropriate credit to the original author(s) and the source, provide a link to the Creative Commons license, and indicate if changes were made.
In cases where reviewers are anonymous, credit should be given to 'Anonymous Referee' and the source.
The images or other third party material in this Peer Review File are included in the article's Creative Commons license, unless indicated otherwise in a credit line to the material. If material is not included in the article's Creative Commons license and your intended use is not permitted by statutory regulation or exceeds the permitted use, you will need to obtain

permission directly from the copyright holder.
To view a copy of this license, visit https://creativecommons.org/licenses/by/4.0/

Response to reviewer's comments

Manuscript: NCOMMS-25-104523

Structural and mechanistic insights into protective non-neutralizing antibodies targeting Crimean-Congo hemorrhagic fever virus Nucleocapsid-protein

We thank the three anonymous reviewers for their insightful and constructive comments. We have addressed all remarks, which are highlighted in blue. Additionally, we provide an updated version of the manuscript file with track changes showing all revisions.

REVIEWER COMMENTS

Reviewer #1 (Remarks to the Author):

In the present manuscript, Moresco, Garrison and colleagues follow up on their previous characterization of anti-CCHFV (Crimean Congo hemorrhagic fever virus) nucleocapsid monoclonal antibodies (mAbs) PMID: 38409240. The current manuscript reports additional mAbs developed at USAMRIID. The authors bin the mAbs to epitopic groups, perform mouse protection studies and report a crystal structure of the 9D5 Fab bound with CCHFV nucleocapsid protein.
Overall, the manuscript offers a first study that correlates epitopes on NP with in vivo protection. It is a relatively straightforward study, but several issues need to be addressed.

We would like to thank Reviewer #1 for their comments and suggestions to improve our manuscript.

Major:

1 – The antibody dose in mouse protection studies is massive. The authors report

1mg/mouse/dose with is approximately 40 mg/kg. Typically, mouse protection experiments do not exceed 20 mg/kg. In the absence of new mouse experiments, the authors should at least clearly state the dose in mg/kg in both the figure legend and the text and discuss the relevance of such a high dose.

The antibody dose given in these studies is consistent with mAb dosing previously used to identify clinically relevant protective CCHFV antibodies by us (manuscript ref. #25,29, 45) and others (PMID: 39002130), which is 50 mg/kg (1 mg per 20 g mouse). Future evaluations will focus on dose down studies and potential combinations. The present study was designed to determine if other anti-NP antibodies conferred any protection in the mouse model. This included whether mAb targeting antigenic sites outside that of 9D5 binding area can also offer protection. These are pre-clinical studies highlighting that not all mAb targeting NP provide comparable protection. Also, that the regions targeted by mAb that confer a level of protection are not neatly siloed to a single domain within the NP. Such understanding is critical for the field to determine optimal dosing, determine developability, and potential synergistic combinations as described in the discussion.

To also address the dosing confusion, we modified the text in the **Methods** and **Results** sections to clearly state the doses given and the rationale. Additionally, *"1 mg per 20 g mouse"* of antibody dose was specified in Fig. 2 legend.

Results: lines 156 to 163

"Recent evidence indicated that humoral immune responses targeting NP are sufficient to confer protection in NP-focused vaccine strategies[39, 40, 41, 46, 47], with at least one anti-NP mAb (mAb-9D5) demonstrating to mediate protective effects[45]. To identify additional non-competing anti-NP mAb within this panel, that may also confer protection when administered prior to CCHFV exposure, the same mouse model previously used for mAb-9D5 was employed."

Methods: lines 473 to 478

"Virus was diluted in a total volume of 0.2 ml PBS. For antibody injections, mice were injected IP with 1 mg per 20 g mouse (50 mg/kg) dose in a total volume of 0.2 ml diluted in PBS. Weights and survival were monitored and plotted using GraphPad Prism 7 software. Since weight loss is typically not an endpoint criterion for CCHFV, the considered euthanasia criteria employed was lethargy and non-responsiveness."

2 – The model of mAb 9D5 binding to the NP oligomer is highly speculative. The authors should perform negative stain EM experiments to show that. Physiologically, a bivalent IgG molecule, as opposed to Fab, would likely bind and therefore disrupt the disrupt the NP oligomer. This should be experimentally tested and discussed in the text.

We apologize to the reviewer for not providing greater clarity around the purpose of Figure 6b. As Reviewer #3 noted, it needed to be reorganized to ensure the intent was clearer to the reader. This intent is to place the finding of the mAb-9D5 epitope in the context of what has already been published and is known in the literature related to structural determination of CCHFV NP oligomeric state (manuscript ref. #35 and 64). We agree with the reviewer that further non-trivial experimentation needs to be done in this field to better understand NPs oligomeric state. Naturally, we would have been scientifically remised if we had not addressed what is currently known in the field in the context of our discoveries.

Hence, the placement of Figure 6b as part of the discussion section of the manuscript. We also updated the title of the figure to ensure there is no confusion on our intent of the figure. More details can be found in our reorganization of this section in our response to Reviewer #3's comments. Out of curiosity, we modeled a full-IgG molecule (using PDBID: 1IGY as a template) and there was no steric hindrance of such a molecule binding. However, as our focus was on the availability of the epitope, we did not want to confuse the reader further with our intentions around Figure 6b.

Minor:

1 – The BLI competition isotherms should be reported in the supplementary data

Thank you for your comment.
Due to the extensive number of isotherm graphs obtained for the binning analyses (~81 combinations total), we added a set of representative raw binning curves exemplifying one run (mAb-5G2 as saturating mAb), with antibodies that show non-competing (mAb-12G10), partially competing (mAb-21B2), and competing binding (mAb-2B11) that equate to the binning values in the charts (Fig. 1a) in the manuscript.

Legend for new Fig S1:
*"**Supplementary Fig. S1.** Saturating mAb-5G2 representative raw isotherm curves showing non-competing (mAb-12G10), partially competing (mAb-21B2), and competing (mAb-2B11) binding. All mAb were introduced to the sensors at 100ug/ml. The graph shows the saturation step followed by an equilibrium/baselining step, followed by a competition step."*

Reviewer #2 (Remarks to the Author):

This manuscript follows the authors' prior work on the monoclonal antibody 9D5 against Crimean-Congo hemorrhagic fever virus nucleoprotein (CCHFV NP). In this study, they attempt to identify more potent anti-NP monoclonal antibodies (mAbs) and classify them into groups using competition assays. They also resolve the complex structure of NP bound to 9D5. While the study addresses an important objective, the results are limited: no mAbs more potent than 9D5 were identified. Moreover, 9D5 itself exhibits only modest in vivo efficacy (~50%-75% protection when administered prophylactically, based on their previous and current studies). The binning analysis does not establish a clear correlation between in vivo protection and binding epitopes, which impairs the overall significance of the work.

We would like to thank Reviewer #2 for their constructive comments. We have carefully considered these and hope that we have improved our manuscript accordingly.

Since structural biology is not my area of expertise, my comments are limited to the biological aspects:

Figure 1—Binning of anti-NP monoclonal antibodies

1. Four colors are used in Figure 1a, but the legend describes only three: pink (≥30% binding inhibition, competition), yellow (30–75%, partial competition), and green (>74%, no competition). What does the cyan color represent?

Thank you for your observation. We double checked the merged pdf and source files used in the review (original Figure 1a below). We did not observe cyan in Figure 1a as highlighted by the reviewer.

Competition mAb

Saturating mAb	mAb	5G2	2B11	2G10	19G9	21B2	12G10	AC06	9D5	BC08
Bin 1	5G2	5	7	-24	34	68	84	68	57	43
	2B11	9	7	-60	32	63	83	63	54	42
Bin 2	2G10	100	106	16	37	111	113	111	114	102
	19G9	101	109	2	24	111	108	108	110	104
Bin 2-3	21B2	82	79	-8	16	14	30	71	59	39
Bin 3-4	12G10	66	84	47	67	4	5	69	63	44
Bin 4	AC06	81	86	45	67	80	87	0	14	7
	9D5	94	101	74	91	96	94	43	8	2
	BC08	98	106	101	107	96	104	66	39	24

.

To address any possible coloring concerns in Fig.1a, we modified the image to choose more color-blind proof colors. Pink for (≥30% binding inhibition, competition), yellow (30–74%, partial competition), and teal (>75%, no competition).

a

Competition mAb

Saturating mAb	mAb	5G2	2B11	2G10	19G9	21B2	12G10	AC06	9D5	BC08
Bin 1	5G2	5	7	-24	34	68	84	68	57	43
	2B11	9	7	-60	32	63	83	63	54	42
Bin 2	2G10	100	106	16	37	111	113	111	114	102
	19G9	101	109	2	24	111	108	108	110	104
Bin 2-3	21B2	82	79	-8	16	14	30	71	59	39
Bin 3-4	12G10	66	84	47	67	4	5	69	63	44
Bin 4	AC06	81	86	45	67	80	87	0	14	7
	9D5	94	101	74	91	96	94	43	8	2
	BC08	98	106	101	107	96	104	66	39	24

Additionally, should the thresholds be defined as 30–74% for partial competition (yellow) and ≥75% for no competition (green)?

Thank you for noticing this. We have corrected Fig.1a legend accordingly to the new version of the figure.

It is also unclear how the competition values were defined/normalized.

Detailed explanation of how the data was normalized was added to the manuscript **Methods section, lines 441 to 454, and to Fig.1a legend.**

"Competition values were normalized as the percent of binding represented by the signal of the competing antibody (Ab2) in the presence of saturating antibody (Ab1) divided by the signal of Ab2 in the absence of Ab1 on the BLI sensor. Ab1 was loaded on the sensor to fully saturate binding before Ab2 was introduced to the sensor."

2. Many competition interactions appear unidirectional, which complicates interpretation. For example:
A. mAb 12G10 (Bin 3–4) shows weak competition (69%/63%/44%) with three mAbs in Bin 4, but no competition is observed in the reverse direction.
B. Similarly, Bin 2–3 shows only one-directional competition with Bin 2.
C. mAb 2G10 (Bin 2) competes strongly with two mAbs from Bin 1 in one direction.

These inconsistencies make the data difficult to interpret and weaken support for the proposed grouping.

Thank you for your comments regarding the epitope binning data. We acknowledge the observation of unidirectional competition, which is a known phenomenon in antibody-antigen interaction studies. For this binning study, the antibodies in the saturating column were loaded on the BLI sensors first to saturation, then the competing antibody was introduced. This is often attributed to differences in antibody affinity, where a high-affinity antibody can displace a lower-affinity antibody, but the reverse is not observed. This is in line with the binding affinities of the mAb (Fig 1b) included in the study.

While these unidirectional results seem inconsistent at first glance, they are often interpretable in the context of differing antibody affinities and do not necessarily invalidate the proposed epitope groupings. The binning assignments were made considering the complete dataset and the order of operations for the saturating *vs* competing mAb, including the strong bidirectional competition observed within the defined bins.

Figure 2—In vivo protective efficacy

1. Although final survival rates for 9D5 and 21B2 were 75% and 50%, respectively, both groups experienced severe weight loss (~20%), indicating only modest protection. None of the newly identified mAbs outperformed 9D5.

Thank you for your comment. Our goal in evaluating *in vivo* protection for other anti-NP mAb besides 9D5 was to identify protective antibodies targeting different regions than 9D5, as discussed in lines 270-285 (original submission). These are pre-clinical studies highlighting that not all mAb targeting NP provide comparable protection. Also, that the regions targeted by mAb that confer a level of protection are not neatly siloed to a single domain within the NP.

2. The IFNAR-/- mouse model is semi-self-limiting for CCHFV infection; survivors after 7 dpi typically clear the virus and regain weight. The ethical endpoint criterion is not specified. If weight loss >30% was used, a more stringent threshold (e.g., >20%) would likely further reduce reported survival rates.

All animal models with an adaptive immune system are self-limiting. The goal is to provide protection until the host adaptive immune response can clear the virus. Weight loss is typically not an endpoint criterion with CCHFV as it is quite transient, and additionally mice often succumb without substantial weight loss. Other mice survive with substantial weight loss and no other indication of disease. Euthanasia criteria are lethargy and non-responsiveness.

The following sentence was added at the **Methods section lines 477 to 478.**
"Since weight loss is typically not an endpoint criterion for CCHFV, the considered euthanasia criteria employed was lethargy and non-responsiveness."

3. Given that survival outcomes depend on the ethical endpoint criteria, additional measures of protection should be provided. These should include viral loads (in liver, spleen, and serum) and histopathological analysis (e.g., H&E staining) of affected tissues.

These additional measurements were in our original 9D5 manuscript (present manuscript ref. #45) and were beyond the scope of this work. The main focus was the structural assessment of 9D5 antibody binding sites. Future studies assessing combinations and optimal dosing will include these additional analyses.

Figure 3—Assignment of Anti-NP mAb epitopes by NP domain

Fig. 3 is organized into subdivisions based on mAb binding epitope areas (bins). For each bin, the NP domain region targeted by the mAb (head or stalk) is indicated in the bottom right-corner of the corresponding panel.

1. Please provide a table listing the KD values for each mAb–NP pair. The binding curve for Bin 4 appears unusual and warrants explanation.

Thank you for your comment. We agree with the reviewer that this topic should be clarified. Although the data presented in Fig. 3 and Fig.1b were both generated using BLI, the objectives of the two assays were distinct. The experiments for the table in Fig1b was performed at numerous concentrations with the goal of specifically calculating the mAb-NP interactions KD and associated data, which rely in the calculation of K_{on} (association) and K_{off} (dissociation) values.

The experiment data shown in Fig. 3 was designed to provide qualitative evidence of mAb binding to the full-length NP and/or the stalk domains by comparing the wavelength shifts (nm) produced by each mAb, relative to the different NP domains. For BLI, the magnitude of the wavelength shift (Y-axis) observed for different mAb binding to full-length NP or NP stalk constructs do not directly correlate with binding affinity, but instead, confirm domain-specific NP binding preferences. Since the intent was not obtaining the KD values, we employed only a short dissociation period which made the Koff calculation not reliable and hence a KD not reliable. As the association event was measured, we did obtain, K_{on} (association) and have provided these in Supplemental Table 1. These experiments were done side-by-side an reveal that the K_{on} rates for an mAb that targets the stalk region has a similar one for the full-length protein.

To minimize potential confusion when comparing kinetic affinity measurements with qualitative binding domains preferences, we highlighted the aims for both assays, and included the detailed methodology employed for the binding kinetics values showed in Fig. 1b.

Results

Subheading line 141: "*Murine-derived anti-NP mAb competition and binding kinetics affinity evaluation"*

Lines 199 to 200: *"MAb-AC06 and mAb-BC08, also belonging to Bin 4, revealed the same full-length NP binding preference, with no noticeable wavelength shifts obtained when NP stalk domain was employed."*

Methods

Lines 456 to 469: *"Binding kinetics of anti-CCHFV NP mAb were measured on an Octet® Red96e system (Sartorius). Full-length recombinant IbAr10200 strain CCHFV NP expressed in SF9 baculovirus cells, (10 µg/ml) was immobilized onto Amine Reactive Second-Generation (AR2G) biosensors (Sartorius) using a standard*

manufacturer's amine coupling kit (Sartorius). Following immobilization and quenching, the NP-loaded sensors were dipped into a baseline 1× kinetics buffer (Sartorius) before being exposed to a serial dilution of anti-CCHFV NP mAb, ranging from 1-100 µg/ml, to monitor the association phase over a 600 s period. Subsequently, the sensors were moved into 1× kinetics buffer alone to measure the dissociation phase over a 1200 s period. Data were analyzed using the Octet® Data Analysis HT software (v. 11.1). The sensorgram data were processed by subtracting the reference channel, aligning the Y-axis to the average of the last 10 s of the baseline step, and applying Savitzky-Golay filtering. The processed curves were then globally fitted to a 1:1 binding model to determine the association rate constant (Kon), dissociation rate constant (Kdis), and the equilibrium dissociation constant (KD).”

Subheading line 479: “*mAb binding preferences to NP distinct domains.*”

We also adjusted Fig. 3 nm scale (Y-axis) together with binding curves thickness to improve visibility and interpretation.

2. I don’t understand why mAbs within the same bin (e.g., Bin 4: 9D5 and AC06, which show similar KD values for Afg09 NP in Figure 1b) exhibit different in vivo protective efficacy.

Thank you for your comment. While Biolayer interferometry (BLI) can identify antigenic sites in respect of one mAb to another, it may not by itself be able to explain complex biologic phenomena, which is a limitation it shares with most assays when viewed in isolation. The bulkiness of Fabs beyond the mAb-antigen interface can prevent mAbs binding to nearby or overlapping sites that may not be compatible with the mode of protection these antibodies provide. We don’t have to look far for an example of this phenomenon. We have shown in previous work (manuscript ref. #29), as well as others (manuscript ref. #62) that different mAb targeting the same CCHFV protein (GP38), belonging to the same bin, and with similar high KD values, do not show the same or similar protective efficacy *in vivo*. X-ray structures showed that while these mAbs are

competitive for the same antigenic site, they have only partially overlapping epitopes, which might explain the differences in protection observed. Similarly, mAb targeting the same CCHFV NP antigenic sites, such as 9D5, AC06, and BC08, can display markedly different or no protection at all. Since mAb-9D5 showed the highest protective efficacy, we reasoned to characterize this relevant epitope (bin 4) to better understand the molecular features of the protective epitope that drives *in vivo* protection.

To clarify this distinction between mAb belonging to the same bin, presenting similar KD values, but distinct protective efficacy, the following sentence was added to the **discussion section, pages 311 to 313.**

"These findings also confirm that different mAb targeting the same antigenic sites (bins) and exhibiting similar binding affinity constants (KD), do not necessarily confer comparable efficacy in vivo[29,62]."

Overall Recommendation
The study presents valuable structural data and a systematic binning analysis. However, the failure to identify superior mAbs and the lack of clear correlation between epitope groups and in vivo protection limit the translational significance of the findings. Including additional virological and pathological data, as noted above, would strengthen the manuscript.

Reviewer #3 (Remarks to the Author):

In this manuscript, Vanessa Moresco et al. identify nine monoclonal antibodies targeting CCHFV NP and systematically characterize their biochemical properties and in vivo protective efficacy. Four epitope bins are defined, among which mAb-9D5, assigned to bin4, confers approximately 75% protection. The authors further determine the structure of the NP-9D5 complex, delineate the precise binding epitope, and, together with sequence analysis, propose a molecular basis for its potential broad protective activity. These findings offer meaningful insights into the functional complexity of CCHFV NP

and provide a valuable foundation for developing antibody-based therapeutics and vaccines. To further strengthen the impact and rigor of this already substantial work, a few aspects could benefit from additional elaboration and contextual discussion.

We would like to thank Reviewer #3 for their supportive comments and suggestions to improve our manuscript.

Major concerns:

1. For Figure 6 and the related discussion, I suggest the authors reorganize their analysis in the context of the current CCHFV RNP structural models. As these existing models are derived from recombinantly expressed proteins (whether solved by X-ray crystallography or EM), the proposed RNP architecture may not represent the authentic viral complex. Consequently, the structural superposition of the NP-Fab complex onto the NP oligomer model carries limited biological significance.

Thank you for this comment and understanding of what may have been our intent with Figure 6b. As suggested, we have reorganized this discussion section to make our intent and the current state of the field clearer to the reader. With the X-ray and Cryo-EM SPA models (manuscript ref. #35 and 64) comprising what is currently known structurally about CCHFV NP oligomerization at the molecular level, the intent of Figure 6b was to discuss how our findings intersect with these previous findings. As the reviewer points out, there is a possibility that these published studies may not completely reflect an oligomerized state of NP within the virus, or the presence of the NPs form on the surface of cells where it was found recently by our recent work (manuscript ref. #45). Among the organization, these points have also been incorporated into the revised text in part by the addition of the below text.

Discussion lines 375 to 378:
"With the absence of a high-ordered CCHFV-NP-RNA structure, neither of these models may be totally reflective of the NPs oligomeric state in the virion. However, the

protective nature of mAb-9D5 attests to the possibility of the epitope binding site to be accessible regardless of the NP conformation present."

Reflecting on the reviewers' comments, we also recognized that the previous title of Figure 6 may have created confusion surrounding the intent of the figure. To avoid further confusion, the title of Figure 6 has been modified to the following:

"mAb-9D5 epitope accessibility related to surfaces implicated in RNA binding and those previously proposed for oligomerization."

2. Line183-188, For epitope mapping, the authors compared antibody binding to the NP stalk region and to the full-length NP protein, and antibodies that bound only to the full-length NP were inferred to recognize the head domain. Could the authors clarify why the head domain itself was not directly tested? Direct binding assays using the isolated head domain would strengthen the conclusions and provide a more straightforward interpretation. A relevant construct design can be found in this literature: Structural and Functional Diversity of Nairovirus-Encoded Nucleoproteins./jvi.01680-15

Sure, we are happy to provide additional insights into why the isolated head domain was not directly tested. The stalk and head domains of NP are not sequentially organized along the peptide chain. This resulted in the head domain including both the N-terminus portion of the protein (residues 1-184), and its C-terminus portion (residues 306-481), while the stalk domain encompasses a segment in the middle of the protein (residues 194-300). Our structural analysis illustrated that simple removal of the C-terminal portion that included the stalk would expose the hydrophobic core of the protein. Such truncations could alter structural integrity and potentially affect how monoclonal antibody (mAb) epitopes are displayed.

This is further supported by a recent study by others (PMID: 41652995), examining T-cell responses to truncate NP protein constructs expressing only the N-terminal, middle, and C-terminal regions. The authors concluded that these truncate forms could limit immunological interpretations, because they would likely be linear.

Although the suggested literature (Structural and Functional Diversity of Nairovirus-Encoded Nucleoproteins./jvi.01680-15) is highly relevant for solving the NP structures for important nairoviruses, only full-length NP constructs were employed to determine these crystallographic structures. Comparisons of individual domains with previously solved CCHFV NP were made by superposing the head and/or stalk domains separately, rather than analyzing isolated domain constructs/structures. In addition, the authors discuss that the isolated head domain for ERVE virus NP was obtained due to spontaneous protein degradation rather than an intentional expression strategy.

3. Among the three antibodies classified into bin4, only 9D5 exhibits protective activity. Given that the other two antibodies compete for the same epitope site, the authors are suggested to explain why these antibodies were not further characterized structurally, as such analyses could provide additional insight into the relationship between this epitope cluster and protective efficacy.

Thank you for your pertinent comment. As we have previously described (manuscript ref. #29), that mAb targeting another CCHFV protein (GP38), belonging to the same bin, and with similar high KD values, not necessarily showed the same or similar protective efficacy *in vivo*. Similarly, mAb targeting the same CCHFV NP epitopes, such as 9D5, AC06, and BC08, can display markedly different or no protection at all. Since mAb-9D5 showed the highest protective efficacy, we focused on the present work in characterizing this relevant epitope (bin 4) in the presence of the most protective mAb to understand the molecular features driving *in vivo* protection.

Minor issue:

1. Line 181-183, while the cited literature covers important aspects of CCHFV NP structures, the background would benefit from including the pioneering work that resolved the first monomeric NP structure and characterized its endonuclease function. Crimean-Congo hemorrhagic fever virus nucleoprotein reveals endonuclease activity in bunyaviruses, Proc. Natl. Acad. Sci. U.S.A., vol. 109, no. 13, pp. 5046–5051, 2012. doi: 10.1073/pnas.1200808109.

Thank you for your suggestion! We added the citation for this relevant CCHFV NP structural work (manuscript ref. #50). We also added language to the discussion to include the location of the endonuclease function to the mAb-9D5 antigenic site. This included an updated Figure 6a with the location of the endonuclease, indicated by the pink ellipse.

2. In Figure 3, the BLI traces appear relatively thick, making it difficult to visualize the binding curve of 9D5 to the NP stalk within bin4. Adjusting the line thickness would improve clarity.

Thank you for your comment. The graphs have been adjusted to improve clarity and visibility.

3. In Figures 4b-d, hydrogen bonds should be explicitly labeled to allow clear distinction from hydrophobic interactions.

Thank you for your suggestion. We have now identified and highlighted relevant hydrogen bonds (shown as black dashes) in Fig. 4.

4. The order of the subpanels in Figure 4 should match their order of appearance in the main text. In addition, the figure legends use capital letters whereas the in-text and figure labels are lowercase; please ensure consistency with the journal's formatting requirements.

Thank you for pointing this out. We have updated the order of the Fig. 4 subpanels so that they match the sequence in the text. We also corrected all figure legends in the manuscript using lowercase lettering to remain consistent with the labels shown in the figures.

5.Lines 226-227, Please note that the manuscript uses both en dashes (–) and em

dashes (—) inconsistently. The authors are suggested to ensure consistency throughout and use the correct dash for each function.

We modified the previous comment to make the text style more consistent.

Response to reviewer's comments

Manuscript: NCOMMS-25-104523

Structural and mechanistic insights into protective non-neutralizing antibodies targeting Crimean-Congo hemorrhagic fever virus nucleocapsid protein

We thank the three anonymous reviewers for their insightful and constructive comments. We have addressed all remarks, which are highlighted in blue. Additionally, we provide an updated version of the manuscript file with track changes showing all revisions.

REVIEWER COMMENTS

Reviewer #1 (Remarks to the Author):

In the present manuscript, Moresco, Garrison and colleagues follow up on their previous characterization of anti-CCHFV (Crimean Congo hemorrhagic fever virus) nucleocapsid monoclonal antibodies (mAbs) PMID: 38409240. The current manuscript reports additional mAbs developed at USAMRIID. The authors bin the mAbs to epitopic groups, perform mouse protection studies and report a crystal structure of the 9D5 Fab bound with CCHFV nucleocapsid protein.
Overall, the manuscript offers a first study that correlates epitopes on NP with in vivo protection. It is a relatively straightforward study, but several issues need to be addressed.

We would like to thank Reviewer #1 for their comments and suggestions to improve our manuscript.

Major:

1 – The antibody dose in mouse protection studies is massive. The authors report 1mg/mouse/dose with is approximately 40 mg/kg. Typically, mouse protection

experiments do not exceed 20 mg/kg. In the absence of new mouse experiments, the authors should at least clearly state the dose in mg/kg in both the figure legend and the text and discuss the relevance of such a high dose.

The antibody dose given in these studies is consistent with mAb dosing previously used to identify clinically relevant protective CCHFV antibodies by us (manuscript ref. #25,29, 45) and others (PMID: 39002130), which is 50 mg/kg (1 mg per 20 g mouse). Future evaluations will focus on dose down studies and potential combinations. The present study was designed to determine if other anti-NP antibodies conferred any protection in the mouse model. This included whether mAb targeting antigenic sites outside that of 9D5 binding area can also offer protection. These are pre-clinical studies highlighting that not all mAb targeting NP provide comparable protection. Also, that the regions targeted by mAb that confer a level of protection are not neatly siloed to a single domain within the NP. Such understanding is critical for the field to determine optimal dosing, determine developability, and potential synergistic combinations as described in the discussion.

To prevent confusion, we modified the text in the **Methods** and **Results** sections to clearly state the doses given and the rationale. Additionally, *"1 mg per 20 g mouse"* of antibody dose was specified in Fig. 2 legend.

Methods: lines 418 to 422

"Virus was diluted in a total volume of 0.2 ml PBS. For antibody injections, mice were injected IP with 1 mg per 20 g mouse (50 mg/kg) dose in a total volume of 0.2 ml diluted in PBS. Weights and survival were monitored and plotted using GraphPad Prism 7 software. Since weight loss is typically not an endpoint criterion for CCHFV, the considered euthanasia criteria employed was lethargy and non-responsiveness."

Results: lines 156 to 160

"Recent evidence indicated that humoral immune responses targeting NP are sufficient to confer protection in NP-focused vaccine strategies[39, 40, 41, 46, 47], with at least one anti-

NP mAb (mAb-9D5) demonstrating to mediate protective effects[45]. To identify additional non-competing anti-NP mAb within this panel, that may also confer protection when administered prior to CCHFV exposure, the same mouse model previously used for mAb-9D5 was employed."

2 – The model of mAb 9D5 binding to the NP oligomer is highly speculative. The authors should perform negative stain EM experiments to show that. Physiologically, a bivalent IgG molecule, as opposed to Fab, would likely bind and therefore disrupt the disrupt the NP oligomer. This should be experimentally tested and discussed in the text.

We apologize to the reviewer for not providing greater clarity around the purpose of Fig. 6b. As Reviewer #3 noted, it needed to be reorganized to ensure the intent was clearer to the reader. This intent is to place the finding of the mAb-9D5 epitope in the context of what has already been published and is known in the literature related to structural determination of CCHFV NP oligomeric state (manuscript ref. #35 and 64). We agree with the reviewer that further non-trivial experimentation needs to be done in this field to better understand NPs oligomeric state. Naturally, we would have been scientifically remised if we had not addressed what is currently known in the field in the context of our discoveries.

Hence, the placement of Fig. 6b as part of the discussion section of the manuscript. We also updated the title of the figure to ensure there is no confusion on our intent of the figure. More details can be found in our reorganization of this section in our response to Reviewer #3's comments. Out of curiosity, we modeled a full-IgG molecule (using PDBID: 1IGY as a template) and there was no steric hindrance of such a binding molecule. However, as our focus was on the availability of the epitope, we did not want to confuse the reader further with our intentions around Fig. 6b.

Minor:

1 – The BLI competition isotherms should be reported in the supplementary data

Thank you for your comment.
Due to the extensive number of isotherm graphs obtained for the binning analyses (~81 combinations total), we added a set of representative raw binning curves exemplifying one run (mAb-5G2 as saturating mAb), with antibodies that show non-competing (mAb-12G10), partially competing (mAb-21B2), and competing binding (mAb-2B11) that equate to the binning values in the charts (Fig. 1a) in the manuscript.

Legend for new Fig. S1:
*“**Supplemental Fig. S1.** Saturating mAb-5G2 representative raw isotherm curves showing non-competing (mAb-12G10), partially competing (mAb-21B2), and competing (mAb-2B11) binding. All mAb were introduced to the sensors at 100ug/ml. The graph shows the saturation step followed by an equilibrium/baselining step, followed by a competition step.”*

Reviewer #2 (Remarks to the Author):

This manuscript follows the authors' prior work on the monoclonal antibody 9D5 against Crimean-Congo hemorrhagic fever virus nucleoprotein (CCHFV NP). In this study, they attempt to identify more potent anti-NP monoclonal antibodies (mAbs) and classify them into groups using competition assays. They also resolve the complex structure of NP bound to 9D5. While the study addresses an important objective, the results are limited: no mAbs more potent than 9D5 were identified. Moreover, 9D5 itself exhibits only modest in vivo efficacy (~50%-75% protection when administered prophylactically, based on their previous and current studies). The binning analysis does not establish a clear correlation between in vivo protection and binding epitopes, which impairs the overall significance of the work.

We would like to thank Reviewer #2 for their constructive comments. We have carefully considered these and hope that we have improved our manuscript accordingly.

Since structural biology is not my area of expertise, my comments are limited to the biological aspects:

Figure 1—Binning of anti-NP monoclonal antibodies

1. Four colors are used in Figure 1a, but the legend describes only three: pink (≥30% binding inhibition, competition), yellow (30–75%, partial competition), and green (>74%, no competition). What does the cyan color represent?

Thank you for your observation. We double checked the merged pdf and source files used in the review (original Fig. 1a below). We did not observe cyan in Fig. 1a as highlighted by the reviewer.

Competition mAb

Saturating mAb	mAb	5G2	2B11	2G10	19G9	21B2	12G10	AC06	9D5	BC08
Bin 1	5G2	5	7	-24	34	68	84	68	57	43
	2B11	9	7	-60	32	63	83	63	54	42
Bin 2	2G10	100	106	16	37	111	113	111	114	102
	19G9	101	109	2	24	111	108	108	110	104
Bin 2-3	21B2	82	79	-8	16	14	30	71	59	39
Bin 3-4	12G10	66	84	47	67	4	5	69	63	44
Bin 4	AC06	81	86	45	67	80	87	0	14	7
	9D5	94	101	74	91	96	94	43	8	2
	BC08	98	106	101	107	96	104	66	39	24

To address any possible coloring concerns in Fig.1a, we modified the image to choose more color-blind proof colors. Pink for (≥30% binding inhibition, competition), yellow (30–74%, partial competition), and teal (>75%, no competition).

a

Competition mAb

Saturating mAb	mAb	5G2	2B11	2G10	19G9	21B2	12G10	AC06	9D5	BC08
Bin 1	5G2	5	7	-24	34	68	84	68	57	43
	2B11	9	7	-60	32	63	83	63	54	42
Bin 2	2G10	100	106	16	37	111	113	111	114	102
	19G9	101	109	2	24	111	108	108	110	104
Bin 2-3	21B2	82	79	-8	16	14	30	71	59	39
Bin 3-4	12G10	66	84	47	67	4	5	69	63	44
Bin 4	AC06	81	86	45	67	80	87	0	14	7
	9D5	94	101	74	91	96	94	43	8	2
	BC08	98	106	101	107	96	104	66	39	24

Additionally, should the thresholds be defined as 30–74% for partial competition (yellow) and ≥75% for no competition (green)?

Thank you for noticing this. We have corrected Fig.1a legend accordingly to the new version of the figure.

It is also unclear how the competition values were defined/normalized.

Thank you for your comments regarding the epitope binning data. The binning assignments were made considering the complete dataset and the order of operations for the saturating *vs* competing mAb, including the strong bidirectional competition observed within the defined bins.

Detailed explanation of how the data was normalized was added to the manuscript **Methods section, lines 395 to 398, and to Fig.1a legend.**
"*Competition values were normalized as the percent of binding represented by the signal of the competing antibody (Ab2) in the presence of saturating antibody (Ab1) divided by the signal of Ab2 in the absence of Ab1 on the BLI sensor. Ab1 was loaded on the sensor to fully saturate binding before Ab2 was introduced to the sensor.*"

Additionally, as suggested by Reviewer #1, we added a set of representative raw binning curves exemplifying one run (mAb-5G2 as saturating mAb), with antibodies that show non-competing (mAb-12G10), partially competing (mAb-21B2) (Supplemental Fig. S1).

2. Many competition interactions appear unidirectional, which complicates interpretation. For example:
A. mAb 12G10 (Bin 3–4) shows weak competition (69%/63%/44%) with three mAbs in Bin 4, but no competition is observed in the reverse direction.
B. Similarly, Bin 2–3 shows only one-directional competition with Bin 2.
C. mAb 2G10 (Bin 2) competes strongly with two mAbs from Bin 1 in one direction.

These inconsistencies make the data difficult to interpret and weaken support for the proposed grouping.

We thank Reviewer #2 for identifying the inconsistency in bin assignment. Following a thorough reanalysis of the competition assay data, we established that only antibodies meeting the total competition criteria (highlighted in pink in Fig. 1a) would be considered in bin classification. The revised bin assignments have been updated accordingly in the Results section and in Fig. 1a. Importantly, the updated classification is now consistent with the head–stalk binding patterns presented in Fig. 3, which has also been revised to reflect these changes. We also updated Fig. 2 to reflect this change.

Regarding the observation of unidirectional competition, we acknowledge that this is a known phenomenon in antibody-antigen interaction studies (Durie et al., Nat. Communications 2022). This is often attributed to differences in antibody affinity, where a high-affinity antibody can displace a lower-affinity antibody, but the reverse is not observed. This is in line with the binding affinities (KD) of the mAb (Fig. 1b) included in the study. While these unidirectional results seem inconsistent at first glance, they are often interpretable in the context of differing antibody affinities and do not necessarily invalidate the proposed epitope groupings. We have previously reported the same methodology for epitope binning via BLI (Golden et al., 2019) and surpasses to what others that have reported their findings in a similar manner via SPR (Pahmeier et al., 2025).

Also, it is important to note that the concept of antibody/epitope binning shown here is challenging to investigate biophysically given the techniques available and the relatively small size of the nucleoprotein antigen relative to the binding antibodies themselves. This dynamic creates an increase likelihood of steric interference between mAbs binding to between antigenic sites.

The following paragraphs were also modified to better explain the reviewed adopted mAb bins classification and its correlation with the binding-domain preferences shown in Fig. 3:

Results section lines 145 to 151: "*MAb-AC06 and mAb-BC08, directly competed with mAb-9D5 and were assigned to Bin 4. MAb-5G2 and mAb-2B11 (Bin 1), mAb-19G9 (Bin 2) and mAb-12G10 (Bin 3) showed no or only partial competition with Bin 4 mAb and were each classified into single bins. In contrast, mAb-2G10 and mAb-21B2 exhibited overlapping competition profiles - mAb-2G10 with Bins 1 and 2, and mAb-21B2 with Bins 2 and 3 - and were thus assigned to shared bins, Bins1-2 and Bins 2-3, respectively (Fig. 1a and Supplemental Fig.1)."*

Results section lines 192-205: "*MAb-AC06 and mAb-BC08, also belonging to Bin 4, and mAb-12G10 (Bin 3) revealed the same full-length NP binding preference, with no noticeable wavelength shifts obtained when NP stalk domain was employed. In contrast, mAb assigned to Bin 1 (5G2 and 2B11) and Bin 2 (19G9) demonstrated similar binding capabilities to the full-length NP while also recognized the isolated stalk domain, indicating that their epitopes localize to the stalk or involve interfaces in which the stalk constitutes a major component. Consistently, mab-2G10 (Bins 1-2) and mAb-21B2 (Bins 2-3) displayed binding profiles similar to those observed for mAb in Bins 1 and 2, and Bins 2 and 3, respectively (Fig. 3). The domain-binding preferences of mab-2G10 and mAb-21B2, together with their bins assignments (Fig. 1a), suggest that the observed competition patterns primarily reflect steric hindrance rather than complete epitope overlap. These findings support a refined binning classification, particularly for relatively small antigens where steric effects can confound interpretations of epitope proximity. This phenomenon may also account for the observed unidirectional blocking behavior in the competition assay."*

Figure 2—In vivo protective efficacy

1. Although final survival rates for 9D5 and 21B2 were 75% and 50%, respectively, both groups experienced severe weight loss (~20%), indicating only modest protection. None of the newly identified mAbs outperformed 9D5.

Thank you for your comment. Our goal in evaluating *in vivo* protection for other anti-NP mAb besides 9D5 was to identify protective antibodies targeting different regions than 9D5, as discussed in lines 270-285 (original submission). These are pre-clinical studies highlighting that not all mAb targeting NP provide comparable protection. Also, that the regions targeted by mAb that confer a level of protection are not neatly siloed to a single domain within the NP.

2. The IFNAR-/- mouse model is semi-self-limiting for CCHFV infection; survivors after 7 dpi typically clear the virus and regain weight. The ethical endpoint criterion is not specified. If weight loss >30% was used, a more stringent threshold (e.g., >20%) would likely further reduce reported survival rates.

All animal models with an adaptive immune system are self-limiting. The goal is to provide protection until the host adaptive immune response can clear the virus. Weight loss is typically not an endpoint criterion with CCHFV as it is quite transient, and additionally mice often succumb without substantial weight loss. Other mice survive with substantial weight loss and no other indication of disease. Euthanasia criteria are lethargy and non-responsiveness.

The following sentence was added at the **Methods section lines 420 and 421.**
"Since weight loss is typically not an endpoint criterion for CCHFV, the considered euthanasia criteria employed was lethargy and non-responsiveness."

3. Given that survival outcomes depend on the ethical endpoint criteria, additional measures of protection should be provided. These should include viral loads (in liver, spleen, and serum) and histopathological analysis (e.g., H&E staining) of affected tissues.

These additional measurements were in our original 9D5 manuscript (present manuscript ref. #45) and were beyond the scope of this work. The main focus was the structural assessment of 9D5 antibody binding sites. Future studies assessing combinations and optimal dosing will include these additional analyses.

Figure 3—Assignment of Anti-NP mAb epitopes by NP domain

Fig. 3 is organized into subdivisions based on mAb binding epitope areas (bins). For each bin, the NP domain region targeted by the mAb (head or stalk) is indicated in the bottom right-corner of the corresponding panel.

1. Please provide a table listing the KD values for each mAb–NP pair. The binding curve for Bin 4 appears unusual and warrants explanation.

Thank you for your comment. We agree with the reviewer that this topic should be clarified. Although the data presented in Fig. 3 and Fig.1b were both generated using BLI, the objectives of the two assays were distinct. The experiments for the table in Fig. 1b was performed at numerous concentrations with the goal of specifically calculating the mAb-NP interactions KD and associated data, which rely in the calculation of K_{on} (association) and K_{off} (dissociation) values.
The experiment data shown in Fig. 3 was designed to provide qualitative evidence of mAb binding to the full-length NP and/or the stalk domains by comparing the wavelength shifts (nm) produced by each mAb, relative to the different NP domains. For BLI, the magnitude of the wavelength shift (Y-axis) observed for different mAb binding to full-length NP or NP stalk constructs do not directly correlate with binding affinity, but instead, confirm domain-specific NP binding preferences. Since the intent was not obtaining the KD values, we employed only a short dissociation period which made the Koff calculation not reliable and hence a KD not reliable. As the association event was measured, we did obtain K_{on} (association) and have provided these in Supplemental Table 1. These experiments were done side-by-side and reveal that the K_{on} rates for an mAb that targets the stalk region has a similar one for the full-length protein.

To minimize potential confusion when comparing kinetic affinity measurements with qualitative binding domains preferences, we highlighted the aims for both assays, and

included the detailed methodology employed for the binding kinetics values shown in Fig. 1b.

Results

Subheading line 141: “*Murine-derived anti-NP mAb competition and binding kinetics affinity evaluation”*

Lines 192 to 194: *“MAb-AC06 and mAb-BC08, also belonging to Bin 4, revealed the same full-length NP binding preference, with no noticeable wavelength shifts obtained when NP stalk domain was employed.”*

Methods

Lines 399 to 412: *“Binding kinetics of anti-CCHFV NP mAb were measured on an Octet® Red96e system (Sartorius). Full-length recombinant IbAr10200 strain CCHFV NP expressed in SF9 baculovirus cells, (10 µg/ml) was immobilized onto Amine Reactive Second-Generation (AR2G) biosensors (Sartorius) using a standard manufacturer's amine coupling kit (Sartorius). Following immobilization and quenching, the NP-loaded sensors were dipped into a baseline 1× kinetics buffer (Sartorius) before being exposed to a serial dilution of anti-CCHFV NP mAb, ranging from 1-100 µg/ml, to monitor the association phase over a 600 s period. Subsequently, the sensors were moved into 1× kinetics buffer alone to measure the dissociation phase over a 1200 s period. Data were analyzed using the Octet® Data Analysis HT software (v. 11.1). The sensorgram data were processed by subtracting the reference channel, aligning the Y-axis to the average of the last 10 s of the baseline step, and applying Savitzky-Golay filtering. The processed curves were then globally fitted to a 1:1 binding model to determine the association rate constant (Kon), dissociation rate constant (Kdis), and the equilibrium dissociation constant (KD).”*

Subheading line 422: “*mAb binding preferences to NP distinct domains.*”

We also adjusted Fig. 3 nm scale (Y-axis) together with binding curves thickness to improve visibility and interpretation.

2. I don't understand why mAbs within the same bin (e.g., Bin 4: 9D5 and AC06, which show similar KD values for Afg09 NP in Figure 1b) exhibit different in vivo protective efficacy.

Thank you for your comment. While Biolayer interferometry (BLI) can identify antigenic sites in respect of one mAb to another, it may not by itself be able to explain complex biologic phenomena, which is a limitation it shares with most assays when viewed in isolation. The bulkiness of Fabs beyond the mAb-antigen interface can prevent mAbs binding to nearby or overlapping sites that may not be compatible with the mode of protection these antibodies provide. We don't have to look far for an example of this phenomenon. We have shown in previous work (manuscript ref. #29), as well as others (manuscript ref. #62) that different mAb targeting the same CCHFV protein (GP38), belonging to the same bin, and with similar high KD values, do not show the same or similar protective efficacy *in vivo*. X-ray structures showed that while these mAbs are competitive for the same antigenic site, they have only partially overlapping epitopes, which might explain the differences in protection observed. Similarly, mAb targeting the same CCHFV NP antigenic sites, such as 9D5, AC06, and BC08, can display markedly different or no protection at all. Since mAb-9D5 showed the highest protective efficacy, we reasoned to characterize this relevant epitope (bin 4) to better understand the molecular features of the protective epitope that drives *in vivo* protection.

To clarify this distinction between mAb belonging to the same bin, presenting similar KD values, but distinct protective efficacy, the following sentence was added to the **discussion section, pages 297 to 299.**

"These findings also confirm that different mAb targeting the same antigenic sites (bins) and exhibiting similar binding affinity constants (KD), do not necessarily confer comparable efficacy in vivo[29,62]."

Overall Recommendation
The study presents valuable structural data and a systematic binning analysis. However, the failure to identify superior mAbs and the lack of clear correlation between epitope groups and in vivo protection limit the translational significance of the findings. Including additional virological and pathological data, as noted above, would strengthen the manuscript.

Reviewer #3 (Remarks to the Author):

In this manuscript, Vanessa Moresco et al. identify nine monoclonal antibodies targeting CCHFV NP and systematically characterize their biochemical properties and in vivo protective efficacy. Four epitope bins are defined, among which mAb-9D5, assigned to bin4, confers approximately 75% protection. The authors further determine the structure of the NP-9D5 complex, delineate the precise binding epitope, and, together with sequence analysis, propose a molecular basis for its potential broad protective activity. These findings offer meaningful insights into the functional complexity of CCHFV NP and provide a valuable foundation for developing antibody-based therapeutics and vaccines. To further strengthen the impact and rigor of this already substantial work, a few aspects could benefit from additional elaboration and contextual discussion.

We would like to thank Reviewer #3 for their supportive comments and suggestions to improve our manuscript.

Major concerns:

1. For Figure 6 and the related discussion, I suggest the authors reorganize their analysis in the context of the current CCHFV RNP structural models. As these existing models are derived from recombinantly expressed proteins (whether solved by X-ray crystallography or EM), the proposed RNP architecture may not represent the authentic viral complex. Consequently, the structural superposition of the NP-Fab complex onto the NP oligomer model carries limited biological significance.

Thank you for this comment and understanding of what may have been our intent with Fig. 6b. As suggested, we have reorganized this discussion section to make our intent and the current state of the field clearer to the reader. With the X-ray and Cryo-EM SPA models (manuscript ref. #35 and 64) comprising what is currently known structurally about CCHFV NP oligomerization at the molecular level, the intent of Fig. 6b was to discuss how our findings intersect with these previous findings. As the reviewer points out, there is a possibility that these published studies may not completely reflect an oligomerized state of NP within the virus, or the presence of the NPs form on the surface of cells where it was found recently by our recent work (manuscript ref. #45). Among the organization, these points have also been incorporated into the revised text in part by the addition of the text below.

Discussion lines 329 to 332:
"With the absence of a high-ordered CCHFV-NP-RNA structure, neither of these models may be totally reflective of the NPs oligomeric state in the virion. However, the protective nature of mAb-9D5 attests to the possibility of the epitope binding site to be accessible regardless of the NP conformation present."

Reflecting on the reviewers' comments, we also recognized that the previous title of Fig. 6 may have created confusion surrounding the intent of the figure. To avoid further confusion, the title of Fig. 6 has been modified to the following:

"mAb-9D5 epitope accessibility related to surfaces implicated in RNA binding and those previously proposed for oligomerization."

2. Line183-188, For epitope mapping, the authors compared antibody binding to the NP stalk region and to the full-length NP protein, and antibodies that bound only to the full-length NP were inferred to recognize the head domain. Could the authors clarify why the head domain itself was not directly tested? Direct binding assays using the isolated head domain would strengthen the conclusions and provide a more straightforward

interpretation. A relevant construct design can be found in this literature: Structural and Functional Diversity of Nairovirus-Encoded Nucleoproteins./jvi.01680-15

Sure, we are happy to provide additional insights into why the isolated head domain was not directly tested. The stalk and head domains of NP are not sequentially organized along the peptide chain. This resulted in the head domain including both the N-terminus portion of the protein (residues 1-184), and its C-terminus portion (residues 306-481), while the stalk domain encompasses a segment in the middle of the protein (residues 194-300). Our structural analysis illustrated that simple removal of the C-terminal portion that included the stalk would expose the hydrophobic core of the protein. Such truncations could alter structural integrity and potentially affect how monoclonal antibody (mAb) epitopes are displayed.
This is further supported by a recent study by others (PMID: 41652995), examining T-cell responses to truncate NP protein constructs expressing only the N-terminal, middle, and C-terminal regions. The authors concluded that these truncate forms could limit immunological interpretations, because they would likely be linear.
Although the suggested literature (Structural and Functional Diversity of Nairovirus-Encoded Nucleoproteins./jvi.01680-15) is highly relevant for solving the NP structures for important nairoviruses, only full-length NP constructs were employed to determine these crystallographic structures. Comparisons of individual domains with previously solved CCHFV NP were made by superposing the head and/or stalk domains separately, rather than analyzing isolated domain constructs/structures. In addition, the authors discuss that the isolated head domain for ERVE virus NP was obtained due to spontaneous protein degradation rather than an intentional expression strategy.

3. Among the three antibodies classified into bin4, only 9D5 exhibits protective activity. Given that the other two antibodies compete for the same epitope site, the authors are suggested to explain why these antibodies were not further characterized structurally, as such analyses could provide additional insight into the relationship between this epitope cluster and protective efficacy.

Thank you for your pertinent comment. As we have previously described (manuscript ref. #29), that mAb targeting another CCHFV protein (GP38), belonging to the same bin, and with similar high KD values, not necessarily showed the same or similar protective efficacy *in vivo*. Similarly, mAb targeting the same CCHFV NP epitopes, such as 9D5, AC06, and BC08, can display markedly different or no protection at all. Since mAb-9D5 showed the highest protective efficacy, we focused on the present work in characterizing this relevant epitope (bin 4) in the presence of the most protective mAb to understand the molecular features driving *in vivo* protection.

Minor issue:

1. Line 181-183, while the cited literature covers important aspects of CCHFV NP structures, the background would benefit from including the pioneering work that resolved the first monomeric NP structure and characterized its endonuclease function. Crimean-Congo hemorrhagic fever virus nucleoprotein reveals endonuclease activity in bunyaviruses, Proc. Natl. Acad. Sci. U.S.A., vol. 109, no. 13, pp. 5046–5051, 2012. doi: 10.1073/pnas.1200808109.

Thank you for your suggestion! We added the citation for this relevant CCHFV NP structural work (manuscript ref. #50). We also added language to the discussion to include the location of the endonuclease function to the mAb-9D5 antigenic site. This included an updated Figure 6a with the location of the endonuclease, indicated by the pink ellipse.

2. In Figure 3, the BLI traces appear relatively thick, making it difficult to visualize the binding curve of 9D5 to the NP stalk within bin4. Adjusting the line thickness would improve clarity.

Thank you for your comment. The graphs have been adjusted to improve clarity and visibility.

3. In Figures 4b-d, hydrogen bonds should be explicitly labeled to allow clear distinction from hydrophobic interactions.

Thank you for your suggestion. We have now identified and highlighted relevant hydrogen bonds (shown as black dashes) in Fig. 4.

4. The order of the subpanels in Figure 4 should match their order of appearance in the main text. In addition, the figure legends use capital letters whereas the in-text and figure labels are lowercase; please ensure consistency with the journal's formatting requirements.

Thank you for pointing this out. We have updated the order of the Fig. 4 subpanels so that they match the sequence in the text. We also corrected all figure legends in the manuscript using lowercase lettering to remain consistent with the labels shown in the figures.

5.Lines 226-227, Please note that the manuscript uses both en dashes (–) and em dashes (—) inconsistently. The authors are suggested to ensure consistency throughout and use the correct dash for each function.

We modified the previous comment to make the text style more consistent.
